# Performance evaluation of virtually deployed road networks for inter-drone communications

**DOI:** 10.1371/journal.pone.0313400

**Published:** 2024-11-14

**Authors:** Kazuyuki Miyakita, Fuga Sato, Keisuke Nakano

**Affiliations:** Graduate School of Science and Technology, Niigata University, Niigata, Japan; University of Lagos Faculty of Engineering, NIGERIA

## Abstract

This paper considers message delivery through direct communication between drones in an epidemic manner. One method of increasing communication opportunities is to enlarge the communication range. However, this approach is not always possible due to power consumption or equipment limitations. As a novel alternative to this method, we consider setting a virtually deployed road network (VDRN) for drones. By making drones move in a distributed manner along a VDRN, they pass close to each other more frequently, increasing communication opportunities. Through theoretical analysis, we clarify how much a VDRN can improve contact performance. We also investigate how much the use of a VDRN can increase travel time, since moving along such a network can cause detours. By comparing the numerical results for the above two methods of increasing communication opportunities, we examine the required duration of detours for VDRNs to achieve the same level of contact performance as enlarging the communication range.

## 1 Introduction

Epidemic communication is a method of transmitting information by direct wireless communication between mobile nodes as well as by the movement of the nodes themselves. This communication technique is used for delay tolerant networks (DTNs) [1–6]. Epidemic communication can deliver information to distant locations even when the node density is low, so this approach has been actively studied as an effective method of transmitting information during disasters. In this regard, not only pedestrians and vehicles but also aircraft and drones are considered nodes for epidemic communication [7–10].

In the research on epidemic communication among drones or vehicles, introducing special mobile nodes (called ferries) dedicated to epidemic communication has also been investigated [7, 8, 10, 11]. Following a predetermined schedule, ferries travel to multiple points in a service area to collect and distribute information within it. While traveling, they exchange information with each other using epidemic communication. If the movement of each ferry could be predetermined, it would also be possible to predetermine when and where ferries pass each other (i.e., the time and place of meeting) to facilitate communication between them. Therefore, a predetermined schedule is an essential tool in using ferries to improve communication performance.

On the other hand, if drones are not special mobile nodes such as ferries, they tend to move independently and distributedly. In such cases, the movement of a drone cannot always be predetermined. In this paper, we investigate how to improve the communication performance between drones without predetermined schedules. As a measure of communication performance, we concentrate on how frequently drones contact each other, since contact performance is essential for epidemic communication.

Even if the movement of drones cannot be predetermined, they can make frequent contact if they are deployed densely and the communication range of each one is large enough. However, in a worst-case scenario, where the movement of drones cannot be predetermined, their distribution is sparse, and their communication range is small, they rarely contact each other. Such situations may arise during a disaster, and in these cases sharing information is vital. In this paper, we target these situations and investigate how to increase and improve contact among drones.

As mentioned above, extending the communication range is one method to increase points of contact, although this option is not always possible due to power consumption and equipment limitations. As an alternate method of increasing contacts, we propose establishing a virtually deployed road network for drones, called a VDRN, along which drones can easily pass each other. In a drone system using VDRNs, after a drone leaves its starting point, it enters the VDRN from the network’s intersection closest to the starting point, moves along the VDRN to the intersection nearest its destination, and finally exits from that intersection to arrive at its destination.

**Fig 1:**
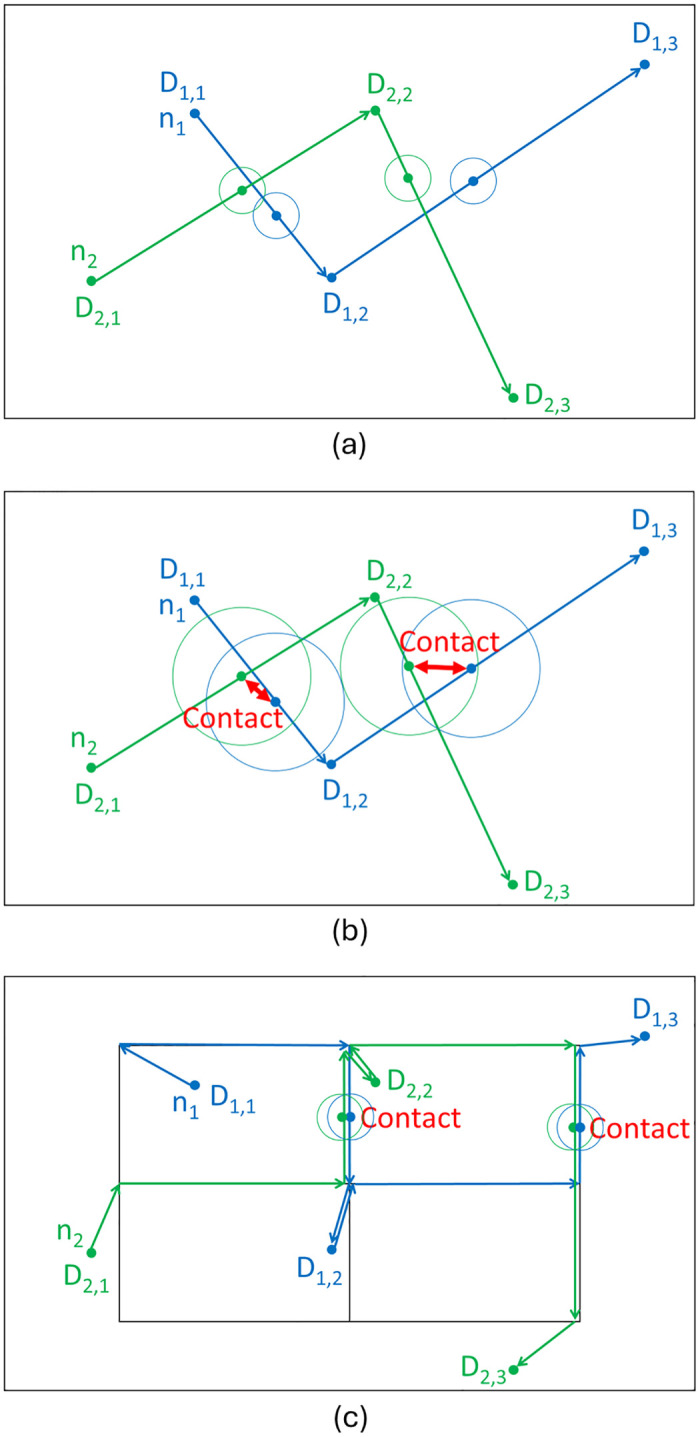
Concept of VDRN. (a) Small communication range, (b) Increased communication range, (c) VDRN.

The concept of our proposal can be explained as follows. Consider the example in Fig 1a. Two drones, *n*_1_ and *n*_2_, travel to their destinations: *D*_1,1_, *D*_1,2_, and *D*_1,3_ for *n*_1_; *D*_2,1_, *D*_2,2_, and *D*_2,3_ for *n*_2_. Here, they move independently of each other. The communication range in Fig 1a is very small relative to the size of the service area. Due to such a limited communication range, *n*_1_ and *n*_2_ do not contact each other during their travels.

Fig 1b shows an example where the communication range increases while maintaining the same destinations of the drones as in Fig 1a. If we used such a large communication range, the communication performance would improve because *n*_1_ and *n*_2_ would contact each other twice.

Fig 1c shows another example of applying the principle of VDRNs. In this case, we consider a VDRN with a lattice structure. The initial positions of *n*_1_ and *n*_2_ and their destinations in Fig 1c are identical to those in Fig 1a and 1b, where these drones move from one destination to the next in a straight line (i.e., with minimum travel time). On the other hand, in Fig 1c, *n*_1_ and *n*_2_ move along the VDRN and take detours of certain lengths. As a result, in this example, *n*_1_ and *n*_2_ pass close together and can then communicate with each other twice. Of course, we can also consider using a VDRN with a different lattice structure than 2 × 2 (essentially, an *N*_*x*_ × *N*_*y*_ lattice structure), since this structure affects contact performance. Here, we assume that drones do not collide because they are designed to take measures that prevent collisions, e.g., using different lanes for return trips on the road or changing altitude.

These examples show that contact performance improves to a greater extent in both Fig 1b and 1c than in Fig 1a. However, this improved contact performance comes at the cost of an increased communication range (Fig 1b) or more detours (Fig 1c). Here, it should be noted that changing the communication range is not easy. In contrast, a VDRN can be freely determined, although this requires a way to inform every drone about the VDRN. In this paper, we investigate how much a VDRN improves contact performance and the duration of detours required to achieve the same level of contact performance as increasing the communication range.

Toward our purpose, we theoretically analyze a VDRN’s contact performance and compare it with the contact performance of non-VDRNs, where drones move directly to their destinations without detours. First, we theoretically analyze the mean contact interval and the mean travel time for non-VDRNs to clarify the relations between these metrics and the communication range; in this work, the former and latter metrics evaluate contact performance and detours, respectively. Next, we theoretically analyze these metrics for a VDRN with a lattice structure and describe the relation between the metrics and the VDRN’s structure. As mentioned above, this paper targets worst-case scenarios, where the distribution of drones is sparse and the communication range is small. In our theoretical analysis, for simplicity, we assume an even more extreme worst-case scenario, where the communication range is extremely small and drones can only communicate with each other when they pass each other on the same virtual road. Finally, we discuss the duration of detours that is required to achieve the same level of contact performance as increasing the communication range.

The rest of this paper is organized as follows. In Section 2, as background information, we present a potential scenario in which the VDRN is used, as well as related works. In Section 3, we clarify the problem statement of this paper, including detailed assumptions about non-VDRNs and VDRNs along with definitions of the performance metrics. In Section 4, we theoretically analyze the mean contact interval and the mean travel time for both non-VDRNs and VDRNs. In Section 5, we show the numerical results of these analyses and explain the characteristics of a VDRN. We also compare the numerical results between non-VDRNs and VDRNs and discuss the duration of detours that is required to achieve the same level of contact performance as could be accomplished by increasing the communication range. Section 6 concludes this paper.

## 2 Background information

### 2.1 Potential scenario: Application to disaster situations

**Fig 2:**
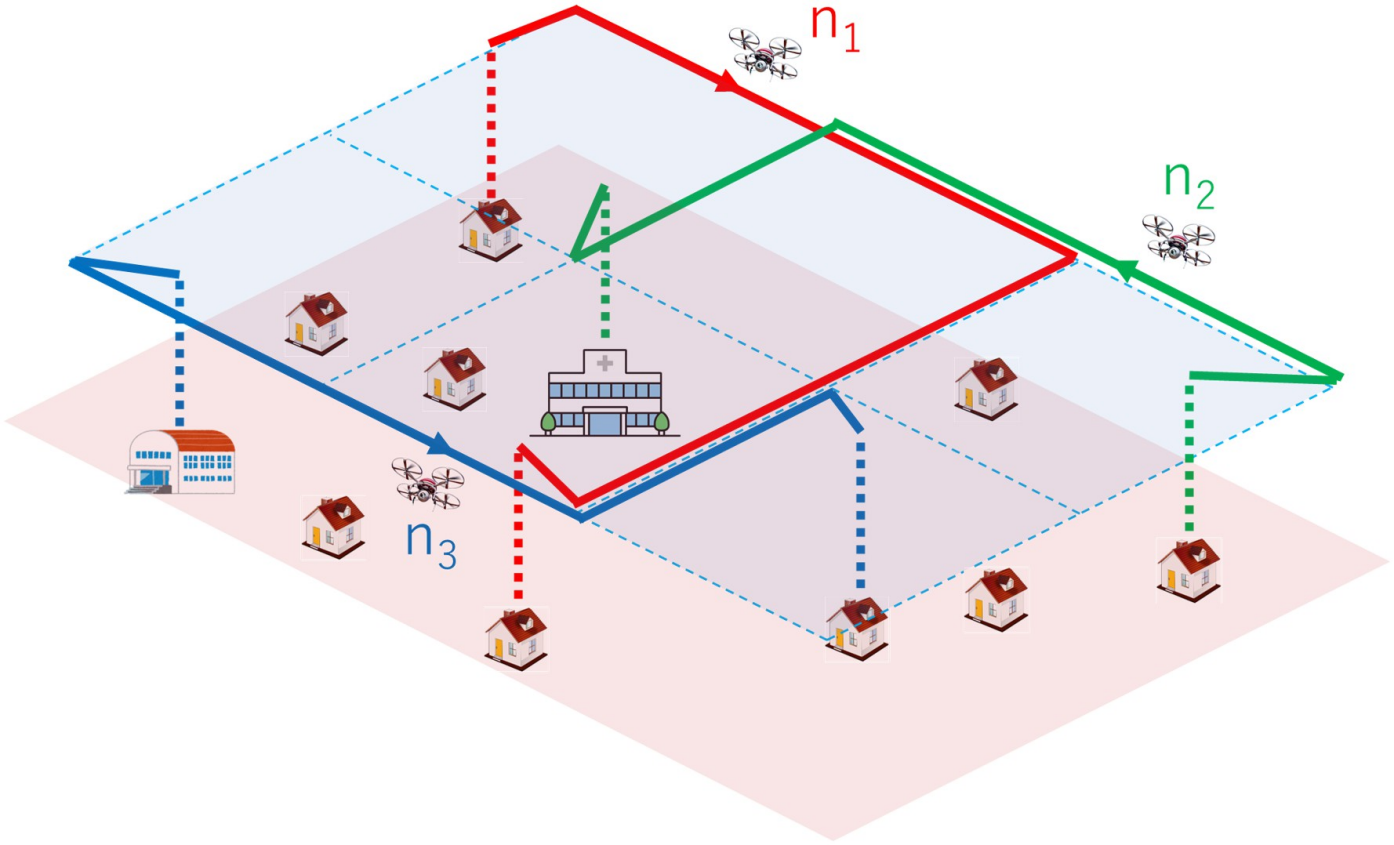
Application of VDRN to disaster scenario.

This subsection describes a potential scenario in which the VDRN is used. Consider a disaster situation like that shown in Fig 2. We assume that the communication infrastructure is disabled due to the disaster. Then we consider the information exchange between evacuation sites, hospitals, and so on using epidemic communication by drones engaged in transporting goods.

In a disaster situation, the transport of goods to evacuation sites is a fundamental task. For bulky goods, conventional vehicles are selected as transporters, while for immediate requests for food and medicine to isolated sites, drones are selected. Here, we consider such transport tasks by drones. Note that these drones are not special mobile nodes such as the ferries discussed in the preceding section; therefore, they basically move independently and distributedly in their transport tasks, not following a predetermined schedule. However, the drones must gather and share various kinds of information to accomplish their transport tasks. Furthermore, as explained below, such communication includes on-demand and real-time information.

First, drones must gather information on what kind of supplies are needed and where they are needed. Note that such information changes with time because it includes on-demand requests for medicine, food etc.; therefore, drones have to update and share such information in real time.

As another kind of real-time information needed to maintain drones, they need to gather information on where they can charge their batteries during travels between evacuation sites. In the event of a disaster, the range of a power outage may be unknown and change with time. Moreover, the condition of each renewable energy supply changes over time due to weather conditions, the amount of demand for supplies, and so on. Accordingly, drones have to gather, update, and share information on charging places in a real-time manner.

With the recent widespread use of drones, it is expected that various types of drones, including both publicly and privately operated drones, will join disaster operations as described above. If all drones can share a predetermined schedule during an operation, the problem is easy; however, it is not easy to estimate future demand in advance. In this paper, therefore, we give attention to drones’ operation using real-time information sharing instead of a predetermined schedule.

In the following, we give some concrete examples:

- If a drone receives a request for medicine at an evacuation site, and this drone is engaged in transport to other places, then it is advantageous for the drone to share this information with other drones that can provide help.
- A drone may encounter a situation in which its battery is drained, a power outage occurs, and there is no location information on available charging places. Even in this case, the drone may be able to obtain information on the location of a renewable energy supply from other drones.

A VDRN helps sharing and updating the above real-time information between drones because it makes them contact each other more frequently. For example, in Fig 2, a VDRN makes it easier for drones *n*_1_, *n*_2_, and *n*_3_, which receive different information from different evacuation sites, to contact each other, and thus it can accomplish quick information exchange. In order for drones to travel along the VDRN, all drones must have a common VDRN map. However, such sharing is less difficult when VDRN maps for various regions are prepared and installed in the drone before a disaster strikes.

### 2.2 Related work

Many papers have addressed the design of predetermined schedules for epidemic communication by drones (i.e., ferries) [12–15]. Unfortunately, unlike the proposed method, these studies have assumed that drones do not move independently and that their movements are predetermined.

In addition, other papers have addressed the design of drone trajectories. For example, reported drone trajectory designs have supported the communications of fixed users on the ground [16–20]. However, these studies do not consider epidemic communication between drones, so they are essentially different in purpose from the approach in this paper. Another previous work [21] proposed a virtually deployed road network for drones, but this system delivered goods and did not assume epidemic communication between drones.

A previous study [22] considered epidemic communication between spacecraft and investigated the optimal information transmission method (i.e., routing), assuming that the trajectory of each spacecraft is known in advance. This study also essentially differs from our aims, since it assumes a predetermined schedule of the spacecraft’s movement.

## 3 Problem statement

In this section, we present our paper’s problem statement. The variables used in this paper are summarized in Table 1.

**Table 1:** Variables used in this study.

Notation	Definition
*a*	horizontal length of service area
*b*	vertical length of service area (*b* ≤ *a*)
*m*	number of drones
*n*_*i*_	*i*th drone (*i* = 1, 2, ⋯, *m*)
*D*_*i*,*j*_	*j*th destination of *n*_*i*_ (*i* = 1, 2, ⋯, *m*, *j* = 1, 2, ⋯)
*v*	speed of each drone
*r*	communication range of each drone
*N*_*x*_	number of road segments in horizontal direction for a VDRN
*N*_*y*_	number of road segments in vertical direction for a VDRN
*d*_*x*_	length of road segments in horizontal direction for a VDRN
*d*_*y*_	length of road segments in vertical direction for a VDRN
*c*_*S*_	closest intersection to *D*_*i*,*j*_ when considering travel from *D*_*i*,*j*_ to *D*_*i*,*j*+1_ for a VDRN
*c*_*D*_	closest intersection to *D*_*i*,*j*+1_ when considering travel from *D*_*i*,*j*_ to *D*_*i*,*j*+1_ for a VDRN
*E*(⋅)	mean of ⋅
*E*(*T*_*c*,*m*_)	mean of time interval in which a drone contacts other drones
*E*(*T*_*c*_)	*E*(*T*_*c*,*m*_) for *m* = 2 (i.e., *E*(*T*_*c*_) = *E*(*T*_*c*,2_))
*E*(*T*_*trip*_)	mean travel time from *D*_*i*,*j*_ to *D*_*i*,*j*+1_
*E*(*T*_*c*,*non*−*VDRN*_)	*E*(*T*_*c*_) for a non-VDRN
*E*(*T*_*trip*,*non*−*VDRN*_)	*E*(*T*_*trip*_) for a non-VDRN
*E*(*T*_*c*,*VDRN*_)	*E*(*T*_*c*_) for a VDRN
*E*(*T*_*trip*,*VDRN*_)	*E*(*T*_*trip*_) for a VDRN

As explained above, the fundamental purpose of introducing VDRN is to increase the contact opportunities for drones. As a performance evaluation, therefore, we concentrate on how much the contact opportunities are increased by VDRN. To simplify the theoretical analysis and clarify the basic characteristics of VDRN, we neglect the complicated factors described for the scenario in Section 2.1 and instead use a simple model as follows.

### 3.1 Basic assumptions about drones

**Fig 3:**
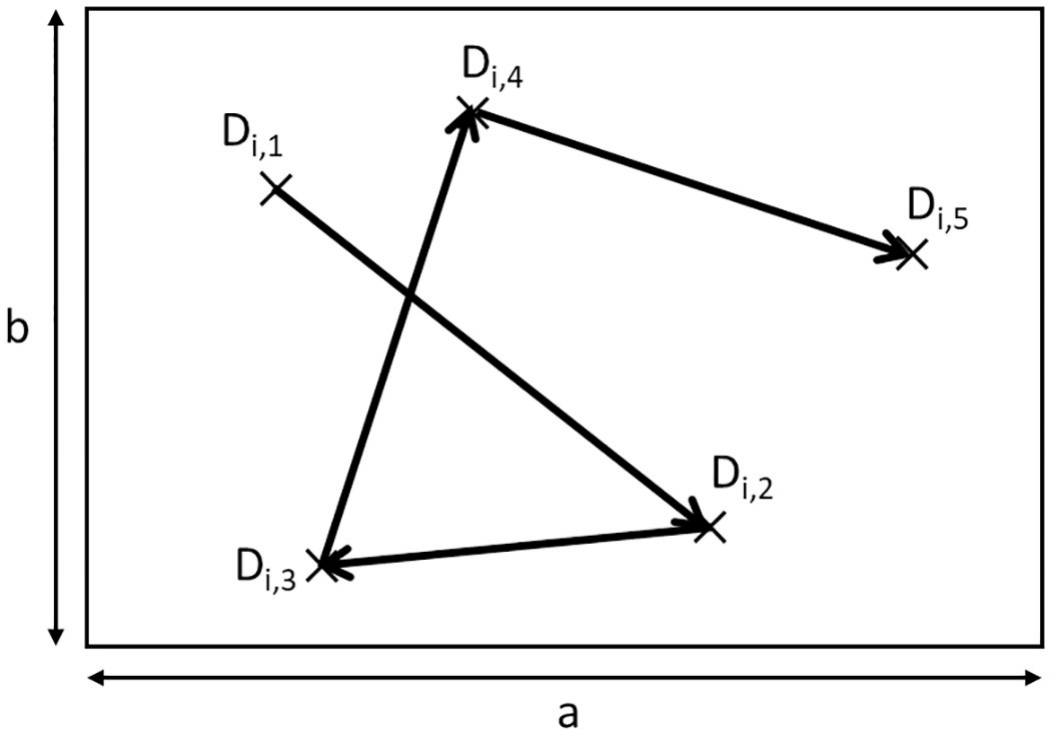
Service area and destinations for drone *n*_*i*_.

We consider a rectangular service area of [0, *a*] × [0, *b*], where *a* ≥ *b*. Suppose *m* drones *n*_1_, *n*_2_, ⋯, *n*_*m*_ are in the service area and that they move independently. Let *D*_*i*,*j*_ be the *j*th destination of drone *n*_*i*_. For all *i* = 1, 2, ⋯, *m* and *j* = 1, 2, ⋯, the position of *D*_*i*,*j*_ is chosen randomly from the service area. An example is shown in Fig 3, where *n*_*i*_ visits *D*_*i*,1_, *D*_*i*,2_, ⋯ in this order.

For simplicity, we neglect the time needed to complete a task at *D*_*i*,*j*_, which is related to the drone’s purpose, and assume that *n*_*i*_ immediately leaves *D*_*i*,*j*_. We also assume that a drone’s flight altitude is constant and neglect the time it takes to rise and descend. The speed of each drone is constant at *v*.

The communication range of each drone is *r*. When two drones enter each other’s communication range, we say that they are in contact with each other, which means that they can communicate with each other.

### 3.2 Assumptions about non-VDRNs

For non-VDRNs, each drone *n*_*i*_ moves in a straight line from *D*_*i*,*j*_ to *D*_*i*,*j*+1_ (Fig 3). This movement model is well-known as the random waypoint (RWP) mobility model [23].

### 3.3 Assumptions about VDRNs

**Fig 4:**
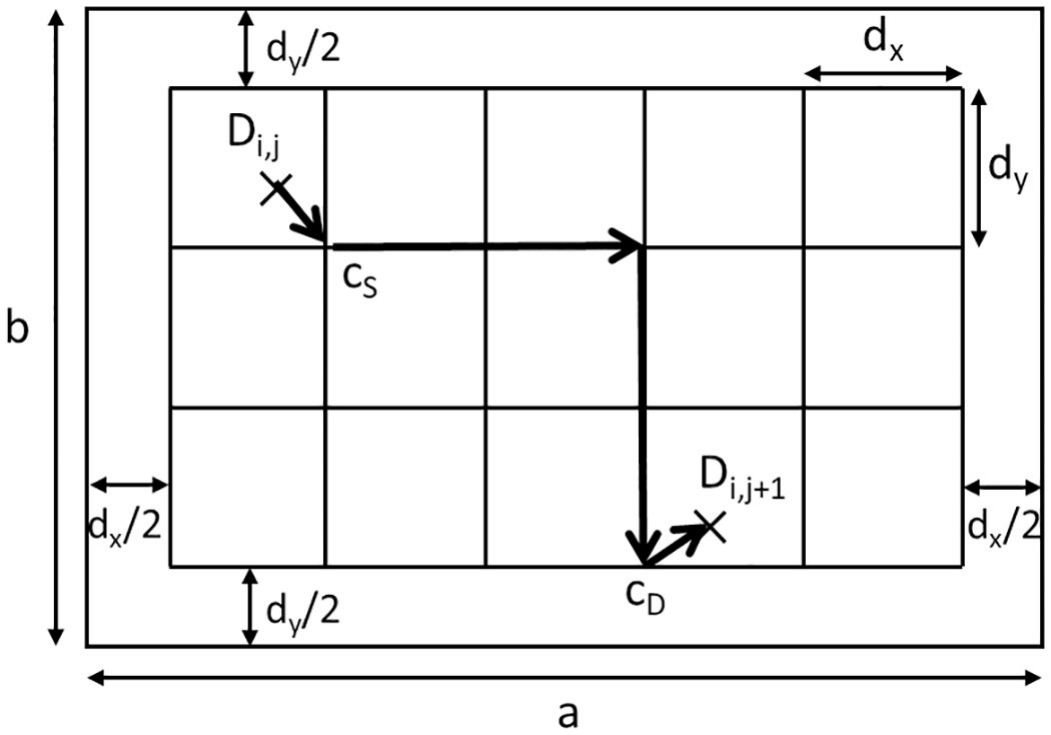
Example of a VDRN where *N*_*x*_ = 5 and *N*_*y*_ = 3.

When using a VDRN, the drones move along it, as shown in Fig 4. In a VDRN, virtual roads are arranged on a lattice of *N*_*x*_ × *N*_*y*_, where *N*_*x*_ and *N*_*y*_ are positive integers. The lengths of the road segments in the horizontal and vertical directions are *d*_*x*_ = *a*/(*N*_*x*_ + 1) and *d*_*y*_ = *b*/(*N*_*y*_ + 1), and the distance between the virtual road and the edge of the service area is *d*_*x*_/2 and *d*_*y*_/2 in the horizontal and vertical directions.

Let *c*_*S*_ and *c*_*D*_ be the closest intersections to *D*_*i*,*j*_ and *D*_*i*,*j*+1_ (Fig 4). A drone moves from *D*_*i*,*j*_ to *c*_*S*_ in a straight line, moves from *c*_*S*_ to *c*_*D*_ along the lattice, and finally moves from *c*_*D*_ to *D*_*i*,*j*+1_ in a straight line. This movement is made repeatedly. This assumption means that a drone moves from *D*_*i*,*j*_ to *c*_*S*_ outside of the VDRN, moves from *c*_*S*_ to *c*_*D*_ along it, and moves from *c*_*D*_ to *D*_*i*,*j*+1_ outside of it.

When moving from *c*_*S*_ to *c*_*D*_ along the lattice, there are several candidate paths for traveling the shortest distance. To simplify the analysis, we assume that a drone moves either first horizontally and then vertically or vice versa. Either case can be chosen with probability 1/2.

For simplicity, this paper assumes that a virtual road is a line with no width and that the altitude of the drones is constant. In practice, however, techniques must be devised to prevent drones from colliding, as mentioned above.

For a VDRN, we assume that *r* is extremely small, which means that the drones contact each other only when they pass each other on the same virtual road.

In this paper, we also assume that all drones have a common VDRN map. To achieve this assumption, we need to prepare such a map and install it on the drones for possible future disasters before actually starting the service. Of course, it would also be desirable to update the VDRN map adaptively to improve performance. The ability to generate and distribute such adaptive VDRN maps remains a future problem.

### 3.4 Performance metrics

For both non-VDRNs and VDRNs, *n*_1_, *n*_2_, ⋯, *n*_*m*_ repeatedly contact each other. Let *T*_*c*,*m*_ be the time interval in which a drone contacts other drones, and let *E*(*T*_*c*,*m*_) be its mean value. *E*(⋅) denotes the mean of ⋅ throughout this paper. A smaller *E*(*T*_*c*,*m*_) is better because that situation provides more communication opportunities between the drones. In this paper, instead of directly analyzing *E*(*T*_*c*,*m*_), we theoretically analyze *E*(*T*_*c*,2_), since *E*(*T*_*c*,*m*_) for any *m* can be easily computed from *E*(*T*_*c*,2_) as *E*(*T*_*c*,*m*_) = *E*(*T*_*c*,2_)/(*m* − 1). Here, we redefine *E*(*T*_*c*,2_) as *E*(*T*_*c*_).

To evaluate the detours caused by moving along a VDRN, we also theoretically analyze the mean travel time from *D*_*i*,*j*_ to *D*_*i*,*j*+1_, denoted by *E*(*T*_*trip*_).

We denote *E*(*T*_*c*_) and *E*(*T*_*trip*_) for a non-VDRN by *E*(*T*_*c*,*non*−*VDRN*_) and *E*(*T*_*trip*,*non*−*VDRN*_) and those for a VDRN by *E*(*T*_*c*,*VDRN*_) and *E*(*T*_*trip*,*VDRN*_). Since in a non-VDRN a drone travels the shortest distance to its destination, we always have *E*(*T*_*trip*,*non*−*VDRN*_) ≤ *E*(*T*_*trip*,*VDRN*_).

In Section 5, instead of directly evaluating *E*(*T*_*c*,*non*−*VDRN*_), we evaluate *E*(*T*_*c*,*non*−*VDRN*_)/*E*(*T*_*trip*,*non*−*VDRN*_) because the value of *E*(*T*_*c*,*non*−*VDRN*_) is usually quite large (e.g., 6000 s), and thus intuitively understanding it is difficult. On the other hand, *E*(*T*_*c*,*non*−*VDRN*_)/*E*(*T*_*trip*,*non*−*VDRN*_) denotes how many trips to destinations are required on average to make a single contact. Consequently, *E*(*T*_*c*,*non*−*VDRN*_)/*E*(*T*_*trip*,*non*−*VDRN*_) is easier to understand than *E*(*T*_*c*,*non*−*VDRN*_). As seen from the analyses in the next section, *E*(*T*_*c*,*non*−*VDRN*_)/*E*(*T*_*trip*,*non*−*VDRN*_) is also represented as a function of only two parameters, *b*/*a* and *r*/*a*, which means that the effects of *v* and the specific values of *a*, *b*, and *r* are eliminated. Namely, if the aspect ratio of the service area is given (i.e., *b*/*a*), we have the same performance regardless of the specific values of *a* and *b*.

In the same manner, to evaluate the contact performance of a VDRN, we assess *E*(*T*_*c*,*VDRN*_)/*E*(*T*_*trip*,*non*−*VDRN*_) instead of *E*(*T*_*c*,*VDRN*_). This value’s meaning is slightly different from the non-VDRN case, since the denominator is not the mean travel time for VDRNs but that for non-VDRNs; however, because the difference is not so large, we use this value to compare VDRNs with non-VDRNs under identical conditions. Furthermore, instead of directly evaluating *E*(*T*_*trip*,*VDRN*_), we calculate *E*(*T*_*trip*,*VDRN*_)/*E*(*T*_*trip*,*non*−*VDRN*_), denoting how many times the mean travel time is increased by moving along a VDRN. Here, *E*(*T*_*c*,*VDRN*_)/*E*(*T*_*trip*,*non*−*VDRN*_) and *E*(*T*_*trip*,*VDRN*_)/*E*(*T*_*trip*,*non*−*VDRN*_) are represented as functions of only three parameters: *b*/*a*, *N*_*x*_, and *N*_*y*_. Therefore, for VDRNs as well, if the aspect ratio of the service area is given (i.e., *b*/*a*), we have the same performance regardless of the specific values of *a* and *b*.

## 4 Analysis

### 4.1 Analysis of *E*(*T*_*trip*,*non*−*VDRN*_)

In this subsection, we show the precise formula of *E*(*T*_*trip*,*non*−*VDRN*_) using a previous work’s results [23].

From the assumptions for non-VDRNs, each drone moves within the service area according to RWP. In this case, mean travel distance *E*(*L*_*non*−*VDRN*_) from *D*_*i*,*j*_ to *D*_*i*,*j*+1_ was precisely analyzed [23]:

$$\begin{array}{cc} E(L_{non-VDRN}) & =\frac{1}{15}\{\frac{a^{3}}{b^{2}}+\frac{b^{3}}{a^{2}}+\sqrt{a^{2}+b^{2}}(3-\frac{a^{2}}{b^{2}}-\frac{b^{2}}{a^{2}})\}\\ & +\frac{1}{6}(\frac{b^{2}}{a}\text{arcosh}\frac{\sqrt{a^{2}+b^{2}}}{b}+\frac{a^{2}}{b}\text{arcosh}\frac{\sqrt{a^{2}+b^{2}}}{a}), \end{array}$$ (1)

where $\text{arcosh}\left(x\right)=\text{log}\left(x+\sqrt{x^{2}-1}\right)$. Using this formula, we have *E*(*T*_*trip*,*non*−*VDRN*_) = *E*(*L*_*non*−*VDRN*_)/*v*.

Note that *E*(*T*_*trip*,*non*−*VDRN*_) does not depend on *r*, since it is also clear from the definition of *E*(*T*_*trip*,*non*−*VDRN*_).

### 4.2 Analysis of *E*(*T*_*c*,*non*−*VDRN*_)

Next, we make an approximate analysis of *E*(*T*_*c*,*non*−*VDRN*_), since its precise analysis is difficult.

We must consider the following two values to analyze *E*(*T*_*c*,*non*−*VDRN*_). The first is the probability that *n*_1_ and *n*_2_ are within communication range of each other at any given time. This probability is denoted by Pr(*R* ≤ *r*), where *R* is a random variable representing the distance between *n*_1_ and *n*_2_. The other value is *E*(*T*_*cc*_), which is the mean time from when *n*_1_ and *n*_2_ enter each other’s communication range to when they leave it. From Pr(*R* ≤ *r*) and *E*(*T*_*cc*_), since we clearly have

$$\begin{array}{c} E\left(T_{c,non-VDRN}\right)=\frac{E(T_{cc})}{\text{Pr}(R\leq r)}, \end{array}$$ (2)

we analyze Pr(*R* ≤ *r*) and *E*(*T*_*cc*_) in the following way.

Pr(*R* ≤ *r*) can be represented as $\text{Pr}\left(R\leq r\right)=\text{Pr}\left(\sqrt{{\left(X_{2}-X_{1}\right)}^{2}+{\left(Y_{2}-Y_{1}\right)}^{2}}\leq r\right)$, where *X*_1_ and *Y*_1_ are the x and y coordinates of *n*_1_ and *X*_2_ and *Y*_2_ are those of *n*_2_. This can also be rewritten as

$$\begin{array}{c} \text{Pr}\left(R\leq r\right)=\text{Pr}\left(\Delta Y\leq r,\Delta X\leq \sqrt{r^{2}-\Delta Y^{2}}\right)=\int_{0}^{r}\int_{0}^{\sqrt{r^{2}-y^{2}}}f_{\Delta X,\Delta Y}\left(x,y\right)dxdy, \end{array}$$ (3)

where Δ*X* = |*X*_2_ − *X*_1_|, Δ*Y* = |*Y*_2_ − *Y*_1_|, and *f*_Δ*X*,Δ*Y*_(*x*, *y*) is the joint probability density function of Δ*X* and Δ*Y*. Because Δ*X* and Δ*Y* are not independent, directly analyzing *f*_Δ*X*,Δ*Y*_(*x*, *y*) is difficult. Therefore, we assume that they are independent. This approximation’s details are shown in Appendix A. From this assumption, we have

$$\begin{array}{c} \text{Pr}\left(R\leq r\right)=\int_{0}^{r}\int_{0}^{\sqrt{r^{2}-y^{2}}}f_{\Delta X}\left(x\right)f_{\Delta Y}\left(y\right)dxdy, \end{array}$$ (4)

where *f*_Δ*X*_(*x*) and *f*_Δ*Y*_(*y*) are the probability density functions of Δ*X* and Δ*Y*. To derive the formulas of *f*_Δ*X*_(*x*) and *f*_Δ*Y*_(*y*), we made further approximations, as shown in Appendix A. As a result, we have

$$\begin{array}{c} f_{\Delta X}\left(x\right)=\{\begin{array}{cc} \frac{12{\left(a-x\right)}^{3}\left(a^{2}+3ax+x^{2}\right)}{5a^{6}}, & 0\leq x\leq a,\\ 0, & \text{otherwise}, \end{array} \end{array}$$ (5)

$$\begin{array}{c} f_{\Delta Y}\left(y\right)=\{\begin{array}{cc} \frac{12{\left(b-y\right)}^{3}\left(b^{2}+3by+y^{2}\right)}{5b^{6}}, & 0\leq y\leq b,\\ 0, & \text{otherwise}. \end{array} \end{array}$$ (6)

We compute Pr(*R* ≤ *r*) by substituting Eqs (5) and (6) into Eq (4). Note that by separately considering Eq (4) in (i) 0 ≤ *r* ≤ *b*, (ii) *b* < *r* ≤ *a*, and (iii) $a<r\leq \sqrt{a^{2}+b^{2}}$, we can represent Eq (4) as a closed form in each of these three cases. However, for space considerations, we omit these three closed forms.

In addition, although the full derivation is shown in Appendix B, we approximately compute *E*(*T*_*cc*_) as

$$\begin{array}{c} E\left(T_{cc}\right)=\frac{\pi^{2}r}{8v}. \end{array}$$ (7)

Finally, we approximately compute *E*(*T*_*c*,*non*−*VDRN*_) by substituting Eqs (4) and (7) into Eq (2).

### 4.3 Analysis of *E*(*T*_*trip*,*VDRN*_)

In this subsection, we derive a precise formula of *E*(*T*_*trip*,*VDRN*_).

Consider node *n*_*i*_ and the *j*th travel of *n*_*i*_ from *D*_*i*,*j*_ to *D*_*i*,*j*+1_. If we use *L*_*VDRN*_ to denote a random variable representing the travel distance from *D*_*i*,*j*_ to *D*_*i*,*j*+1_ along the VDRN, then *E*(*T*_*trip*,*VDRN*_) is represented as *E*(*L*_*VDRN*_)/*v*. Therefore, we analyzed *E*(*L*_*VDRN*_) as shown below.

The *L*_*VDRN*_ components include the distance traveled along the VDRN and the distance traveled outside of it. We denote the former and the latter by *L*_*L*_ and *L*_*NL*_. *L*_*L*_ is the Manhattan distance between *c*_*S*_ and *c*_*D*_, which are the closest intersections to *D*_*i*,*j*_ and *D*_*i*,*j*+1_, and this distance varies based on the positions of *c*_*S*_ and *c*_*D*_. *L*_*NL*_ is the sum of the straight-line distance between *D*_*i*,*j*_ and *c*_*S*_ and that between *c*_*D*_ and *D*_*i*,*j*+1_, and it varies based on how close *D*_*i*,*j*_ and *D*_*i*,*j*+1_ are to the nearest intersections. Using *L*_*L*_ and *L*_*NL*_, *E*(*L*) is expressed as

$$\begin{array}{c} E\left(L_{VDRN}\right)=E\left(L_{L}\right)+E\left(L_{NL}\right). \end{array}$$ (8)

Next we analyzed *E*(*L*_*L*_) and *E*(*L*_*NL*_).

*E*(*L*_*L*_) can be computed by considering all combinations of *c*_*S*_ and *c*_*D*_:

$$\begin{array}{cc} E(L_{L}) & =\frac{\sum_{i_{S}=0}^{N_{x}}\sum_{j_{S}=0}^{N_{y}}\sum_{i_{D}=0}^{N_{x}}\sum_{j_{D}=0}^{N_{y}}\left(\right|i_{S}-i_{D}|d_{x}+|j_{S}-j_{D}\left|d_{y}\right)}{{\left(N_{x}+1\right)}^{2}{\left(N_{y}+1\right)}^{2}}\\ & =\frac{N_{x}\left(N_{x}+2\right)}{3(N_{x}+1)}d_{x}+\frac{N_{y}\left(N_{y}+2\right)}{3(N_{y}+1)}d_{y}. \end{array}$$ (9)

*E*(*L*_*NL*_) is analyzed as follows. Since *D*_*i*,*j*_ is chosen uniformly from the service area, the coordinates of *D*_*i*,*j*_ relative to *c*_*S*_ are uniformly distributed at −*d*_*x*_/2 ≤ *x* < *d*_*x*_/2 and −*d*_*y*_/2 ≤ *y* < *d*_*y*_/2. The distance between *D*_*i*,*j*_ and *c*_*S*_ is $\sqrt{x^{2}+y^{2}}$. Therefore, we can compute its mean value:

$$\begin{array}{c} E\left(L_{NL,1}\right)=\frac{1}{d_{x}d_{y}}\int_{-\frac{d_{x}}{2}}^{\frac{d_{x}}{2}}\int_{-\frac{d_{y}}{2}}^{\frac{d_{y}}{2}}\sqrt{x^{2}+y^{2}}dydx=\frac{2d_{x}d_{y}\alpha +d_{y}^{3}\;\text{log}\;\frac{d_{x}+\alpha }{d_{y}}+d_{x}^{3}\;\text{log}\;\frac{d_{y}+\alpha }{d_{x}}}{12d_{x}d_{y}}, \end{array}$$ (10)

where $\alpha =\sqrt{d_{x}^{2}+d_{y}^{2}}$. The same formula can be used for the distance between *c*_*D*_ and *D*_*i*,*j*+1_. As a result, *E*(*L*_*NL*_), which is twice the value of *E*(*L*_*NL*,1_), is computed by

$$\begin{array}{c} E\left(L_{NL}\right)=\frac{2d_{x}d_{y}\alpha +d_{y}^{3}\;\text{log}\;\frac{d_{x}+\alpha }{d_{y}}+d_{x}^{3}\;\text{log}\;\frac{d_{y}+\alpha }{d_{x}}}{6d_{x}d_{y}}. \end{array}$$ (11)

From the above equations, *E*(*L*_*VDRN*_) can be computed by substituting Eqs (9) and (11) into Eq (8). Finally, *E*(*T*_*trip*,*VDRN*_) can be computed by *E*(*T*_*trip*,*VDRN*_) = *E*(*L*_*VDRN*_)/*v*.

### 4.4 Analysis of *E*(*T*_*c*,*VDRN*_)

**Fig 5:**
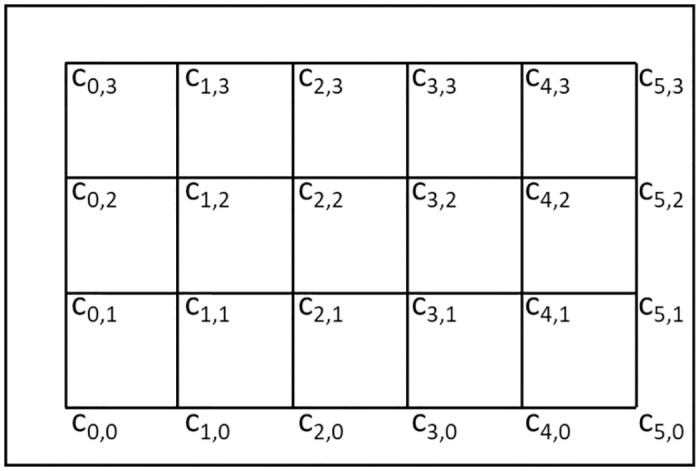
Definition of intersection *c*_*i*,*j*_.

In this subsection, we derive a precise formula of *E*(*T*_*c*,*VDRN*_) by denoting by *c*_*i*,*j*_ an intersection at coordinates (*x*, *y*) = (*id*_*x*_ + *d*_*x*_/2, *jd*_*y*_ + *d*_*y*_/2) for *i* and *j* such that 0 ≤ *i* ≤ *N*_*x*_ and 0 ≤ *j* ≤ *N*_*y*_ (Fig 5). *C* denotes the set of all intersections.

For any time *t* and a very small time duration *dt*, let *dp*_*c*_ be the probability that *n*_1_ and *n*_2_ pass each other between times *t* and *t* + *dt*. Since the mean contact time interval is *E*(*T*_*c*,*VDRN*_), it can be represented as

$$\begin{array}{c} dp_{c}=\frac{dt}{E(T_{c,VDRN})}. \end{array}$$ (12)

On the other hand, *dp*_*c*_ can also be represented as follows. For neighboring intersections P and Q, let PQ be the road segment from P to Q. Let *A*_1_(*PQ*) be an event where a drone is at PQ at any given time. Let *S*_*E*_, *S*_*W*_, *S*_*N*_, and *S*_*S*_ be the sets of all PQs such that Q is the right, left, upper, and lower neighbor of P, respectively. A necessary and sufficient condition for *n*_1_ and *n*_2_ to pass each other between times *t* and *t* + *dt* is that *n*_1_ is in PQ, *n*_2_ is in QP, and at time *t* they are moving toward each other and the distance between them is less than 2*vdt*:

$$\begin{array}{cc} dp_{c}= & \sum_{PQ\in S_{E}\cup S_{W}}\text{Pr}\left(A_{1}\left(PQ\right)\right)\text{Pr}\left(A_{1}\left(QP\right)\right)\frac{2vdt}{d_{x}}\\ & +\sum_{PQ\in S_{N}\cup S_{S}}\text{Pr}\left(A_{1}\left(PQ\right)\right)\text{Pr}\left(A_{1}\left(QP\right)\right)\frac{2vdt}{d_{y}}. \end{array}$$ (13)

From Eqs (12) and (13), we have

$$\begin{array}{cc} E(T_{c,VDRN})=1/ & \{\sum_{PQ\in S_{E}\cup S_{W}}\text{Pr}\left(A_{1}\left(PQ\right)\right)\text{Pr}\left(A_{1}\left(QP\right)\right)\frac{2v}{d_{x}}\\ & +\sum_{PQ\in S_{N}\cup S_{S}}\text{Pr}\left(A_{1}\left(PQ\right)\right)\text{Pr}\left(A_{1}\left(QP\right)\right)\frac{2v}{d_{y}}\}. \end{array}$$ (14)

We compute this by next analyzing Pr(*A*_1_(*PQ*)).

Although the full derivation is shown in Appendix C, Pr(*A*_1_(*PQ*)) for *PQ* ∈ *S*_*E*_ and Pr(*A*_1_(*QP*)) for *QP* ∈ *S*_*W*_ can be computed as

$$\begin{array}{cc} & \text{Pr}\left(A_{1}\left(PQ\right)|PQ\in S_{E}\right)=\text{Pr}\left(A_{1}\left(QP\right)|QP\in S_{W}\right)\\ & =\frac{E(L_{L})}{E\left(L_{L}\right)+E\left(L_{NL}\right)}\cdot \frac{3\left(i_{P}+1\right)\left(N_{x}-i_{P}\right)d_{x}}{{\left(N_{x}+1\right)}^{2}\left(N_{y}+1\right)\{\frac{{\left(N_{x}+1\right)}^{2}-1}{N_{x}+1}d_{x}+\frac{{\left(N_{y}+1\right)}^{2}-1}{N_{y}+1}d_{y}\}}, \end{array}$$ (15)

where $P=c_{i_{P},j_{P}}$ and $Q=c_{i_{P}+1,j_{P}}$. Moreover, Pr(*A*_1_(*PQ*)) for *PQ* ∈ *S*_*N*_ and Pr(*A*_1_(*QP*)) for *QP* ∈ *S*_*S*_ can be computed as

$$\begin{array}{cc} & \text{Pr}\left(A_{1}\left(PQ\right)|PQ\in S_{N}\right)=\text{Pr}\left(A_{1}\left(QP\right)|QP\in S_{S}\right)\\ & =\frac{E(L_{L})}{E\left(L_{L}\right)+E\left(L_{NL}\right)}\cdot \frac{3\left(j_{P}+1\right)\left(N_{y}-j_{P}\right)d_{y}}{\left(N_{x}+1\right){\left(N_{y}+1\right)}^{2}\{\frac{{\left(N_{x}+1\right)}^{2}-1}{N_{x}+1}d_{x}+\frac{{\left(N_{y}+1\right)}^{2}-1}{N_{y}+1}d_{y}\}}, \end{array}$$ (16)

where $P=c_{i_{P},j_{P}}$ and $Q=c_{i_{P},j_{P}+1}$.

Finally, by substituting Eqs (15) and (16) into Eq (14), we obtain the following formula for *E*(*T*_*c*,*VDRN*_):

$$\begin{array}{cc} E(T_{c,VDRN}) & =\{\frac{E\left(L_{L}\right)+E\left(L_{NL}\right)}{E(L_{L})}{\}}^{2}\\ & \times \frac{5\left(N_{x}+1\right)\left(N_{y}+1\right)\{\frac{{\left(N_{x}+1\right)}^{2}-1}{N_{x}+1}d_{x}+\frac{{\left(N_{y}+1\right)}^{2}-1}{N_{y}+1}d_{y}{\}}^{2}}{6v\{\frac{{\left(N_{x}+1\right)}^{4}-1}{{\left(N_{x}+1\right)}^{2}}d_{x}+\frac{{\left(N_{y}+1\right)}^{4}-1}{{\left(N_{y}+1\right)}^{2}}d_{y}\}}. \end{array}$$ (17)

## 5 Numerical results and discussions

### 5.1 Validation of the theoretical analysis for a non-VDRN

**Fig 6:**
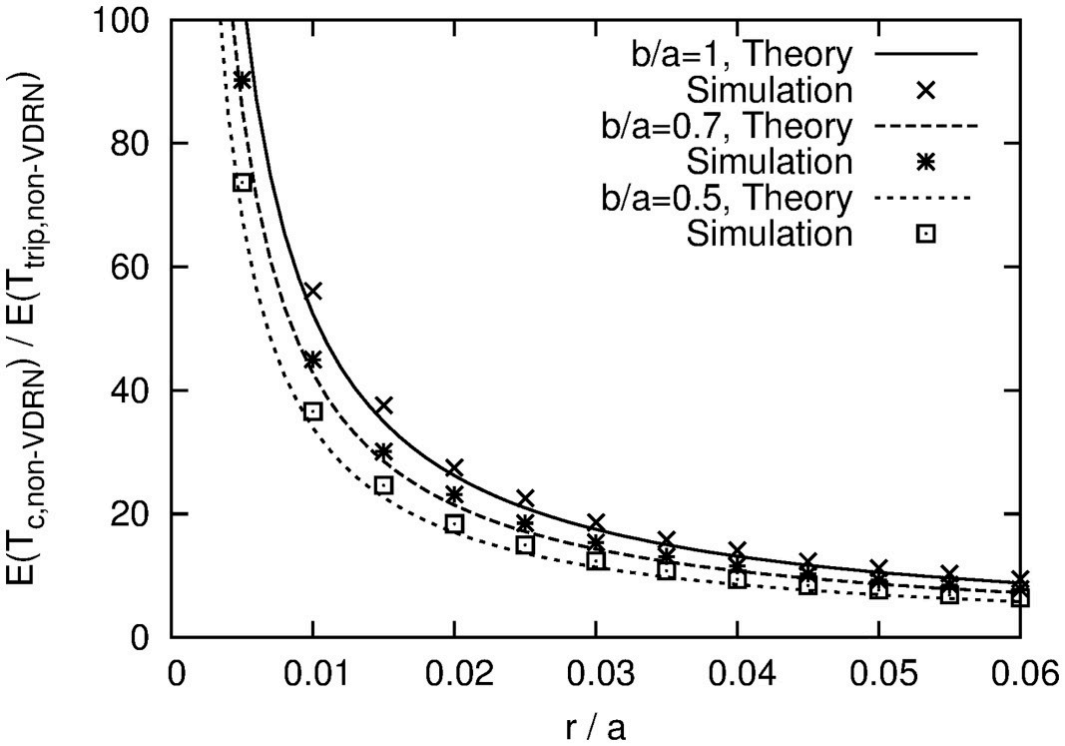
Numerical and simulation results of *E*(*T*_*c*,*non*−*VDRN*_)/*E*(*T*_*trip*,*non*−*VDRN*_).

To confirm the validity of our theoretical analysis of *E*(*T*_*c*,*non*−*VDRN*_), we show the numerical results with the simulation results in Fig 6. This figure shows the results of *E*(*T*_*c*,*non*−*VDRN*_)/*E*(*T*_*trip*,*non*−*VDRN*_) instead of *E*(*T*_*c*,*non*−*VDRN*_). If the numerical and simulation results of *E*(*T*_*c*,*non*−*VDRN*_)/*E*(*T*_*trip*,*non*−*VDRN*_) are close to each other, we can confirm the validity of the theoretical analysis of *E*(*T*_*c*,*non*−*VDRN*_) because *E*(*T*_*trip*,*non*−*VDRN*_)’s theoretical analysis is precise. This figure shows the results for *b*/*a* = 1, 0.7, and 0.5, and the horizontal axis denotes *r*/*a*.

For the simulation, an original simulator was developed by the authors using C#. In this simulator, two drones were deployed in an *a* × *b* rectangular service area where they moved independently based on the assumptions in Sections 3.1 and 3.2. We observed whether the drones were within communication range of each other at a constant time interval of Δ*t* = 0.1 s. Here, we calculated the time intervals at which a drone entered the communication range of another drone and its mean value as *E*(*T*_*c*,*non*−*VDRN*_), and we calculated the mean travel time of the drones as *E*(*T*_*trip*,*non*−*VDRN*_).

The simulation parameters are summarized in Table 2. As mentioned above, we seek a situation in which the communication range is much smaller than the service area. To do this, we set *a* = 10 km and *r* = 50 m, 100 m, ⋯, 600 m. A larger *r* in this range is difficult to achieve by such common wireless systems as Wireless LAN and Bluetooth.

**Table 2:** Parameters used for a non-VDRN simulation.

Parameter	Value
*a*	10 km
*b*	10 km, 7 km, 5 km
*m*	2
*v*	36 km/h
*r*	50 m, 100 m, ⋯, 600 m
Simulation time	10^8^ s

Here, the computation of *E*(*T*_*trip*,*non*−*VDRN*_) is easy, as can be seen from Eq (1). For *E*(*T*_*c*,*non*−*VDRN*_), the formula of Pr(*R* ≤ *r*) includes an integral form. As mentioned, this integral can be represented as closed forms in the three cases given above. Therefore, we can also compute this equation very quickly using the closed forms. The remaining part of Eq (2), other than the integral form, is *E*(*T*_*cc*_) of Eq (7), and this is clearly easy to compute. Furthermore, we can compute *E*(*T*_*trip*,*non*−*VDRN*_) and *E*(*T*_*c*,*non*−*VDRN*_) easily and quickly regardless of parameters *a*, *b*, *v*, and *r*. Moreover, their computation times are much shorter than those of the simulation. Actually, the numerical results of *E*(*T*_*c*,*non*−*VDRN*_)/*E*(*T*_*trip*,*non*−*VDRN*_) in Fig 6 were obtained instantly. For example, Fig 6 has three curves, and these curves consist of 180 points in total. The average computation time of these points is 0.09788 ms by using Wolfram Mathematica version 14.1, running Windows 10 Pro on a typical desktop PC with a 3.20 GHz Intel Core i7 processor and 32 GB RAM. On the other hand, the average computation time of the simulation results of 36 points in Fig 6 is 94.53 s, even though the same PC was used.

Fig 6 shows that for all three values of *b*/*a*, the numerical and simulation results are in good agreement. This implies that the approximation used in the analysis of *E*(*T*_*c*,*non*−*VDRN*_) is reasonable. Therefore, we use the approximate formula of *E*(*T*_*c*,*non*−*VDRN*_) to compare a VDRN and a non-VDRN in Section 5.3.

### 5.2 Fundamental VDRN characteristics

In this subsection, we evaluate the contact performance of a VDRN using *E*(*T*_*c*,*VDRN*_)/*E*(*T*_*trip*,*non*−*VDRN*_) and the resulting detours using *E*(*T*_*trip*,*VDRN*_)/*E*(*T*_*trip*,*non*−*VDRN*_). Similar to the non-VDRN case, we can compute *E*(*T*_*trip*,*VDRN*_) and *E*(*T*_*c*,*VDRN*_) easily and quickly regardless of parameters *a*, *b*, *v*, *N*_*x*_, and *N*_*y*_, since they are represented as closed forms.

**Fig 7:**
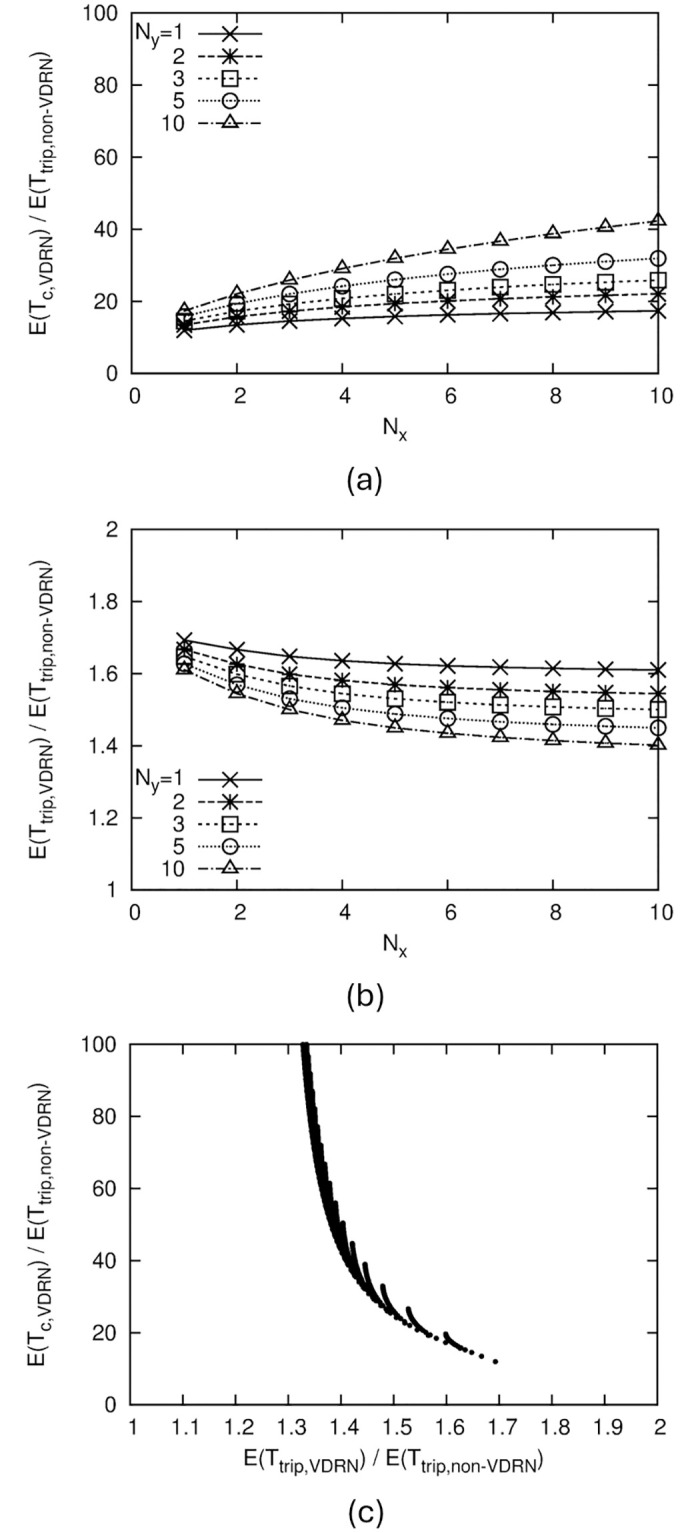
Numerical results of VDRN, where *b*/*a* = 1. (a) Relation between *E*(*T*_*c*,*VDRN*_)/*E*(*T*_*trip*,*non*−*VDRN*_) and VDRN structure, (b) Relation between *E*(*T*_*trip*,*VDRN*_)/*E*(*T*_*trip*,*non*−*VDRN*_) and VDRN structure, (c) Relation between *E*(*T*_*c*,*VDRN*_)/*E*(*T*_*trip*,*non*−*VDRN*_) and *E*(*T*_*trip*,*VDRN*_)/*E*(*T*_*trip*,*non*−*VDRN*_).

Fig 7 shows the numerical results for *b*/*a* = 1. In this figure, we illustrate the effect of *N*_*x*_ and *N*_*y*_ on performance to consider how the VDRN structure affects performance. Fig 7a confirms that dividing the lattice network into smaller sections makes it more difficult for drones to pass each other. On the other hand, such a division also decreases the amount of detours in Fig 7b. These results suggest a trade-off between *E*(*T*_*c*,*VDRN*_) and *E*(*T*_*trip*,*VDRN*_).

To directly show the trade-off between contact performance and detours, we illustrate the relation between *E*(*T*_*c*,*VDRN*_)/*E*(*T*_*trip*,*non*−*VDRN*_) and *E*(*T*_*trip*,*VDRN*_)/*E*(*T*_*trip*,*non*−*VDRN*_) in Fig 7c. Here, the values of *E*(*T*_*c*,*VDRN*_)/*E*(*T*_*trip*,*non*−*VDRN*_) and *E*(*T*_*trip*,*VDRN*_)/*E*(*T*_*trip*,*non*−*VDRN*_) for a pair of *N*_*x*_ and *N*_*y*_ are drawn as a point, and these points for *N*_*x*_ = 1, 2, ⋯, 100 and *N*_*y*_ = 1, 2, ⋯, 100 are shown.

Fig 7c shows that for *b*/*a* = 1, the minimum value of *E*(*T*_*c*,*VDRN*_)/*E*(*T*_*trip*,*non*−*VDRN*_) is about 12. This value indicates the best contact performance that can be achieved by the VDRN for *b*/*a* = 1. We can obtain this value when *N*_*x*_ = 1 and *N*_*y*_ = 1, i.e., a single square-shaped road network. To achieve this performance, the mean travel time is increased by about 1.7 times compared to that without detours.

The contact performance does not improve much in a range of mean travel time less than about 1.4 times. This implies that to improve the contact performance using a VDRN for *b*/*a* = 1, the mean travel time must be at least 1.4 times higher than that without detours.

**Fig 8:**
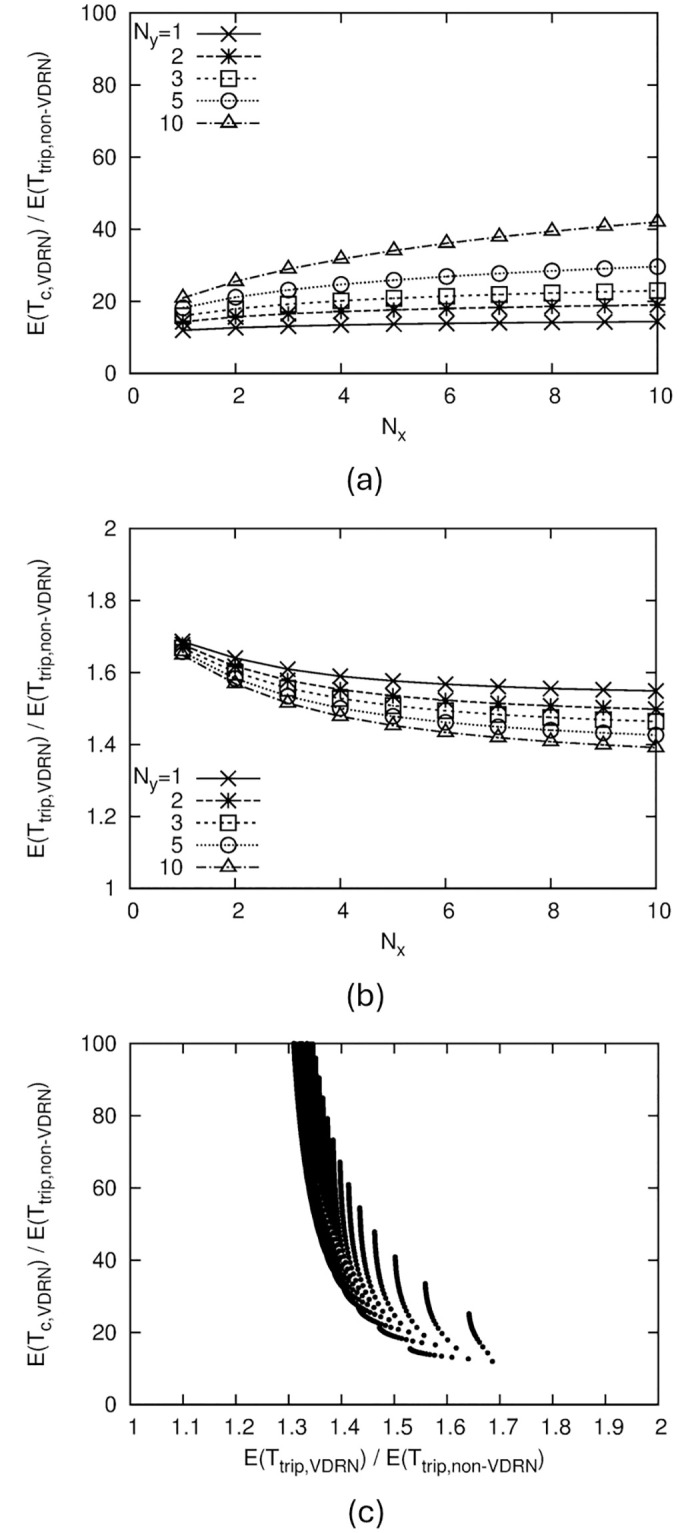
Numerical results of VDRN, where *b*/*a* = 0.7. (a) Relation between *E*(*T*_*c*,*VDRN*_)/*E*(*T*_*trip*,*non*−*VDRN*_) and VDRN structure, (b) Relation between *E*(*T*_*trip*,*VDRN*_)/*E*(*T*_*trip*,*non*−*VDRN*_) and VDRN structure, (c) Relation between *E*(*T*_*c*,*VDRN*_)/*E*(*T*_*trip*,*non*−*VDRN*_) and *E*(*T*_*trip*,*VDRN*_)/*E*(*T*_*trip*,*non*−*VDRN*_).

Fig 8 shows the *b*/*a* = 0.7 results. We found the same tendency as that for *b*/*a* = 1.

### 5.3 Comparison between non-VDRNs and VDRNs

Next, we discuss the relationship between contact performances and the detours for a non-VDRN and a VDRN.

First, recall Fig 6 and consider, as an example, the case where *b*/*a* = 1 and *r*/*a* = 0.01. Value *r*/*a* = 0.01 assumes a very small communication range compared to the size of the service area; such a small *r*/*a* is our target. In this case, *E*(*T*_*c*_)/*E*(*T*_*trip*,*non*−*VDRN*_) is about 60. This value is quite large and implies a very low contact performance, since the frequency of contact is such that a drone can make one contact after traveling to about 60 destinations. Therefore, we must reduce this value.

If we use a VDRN, we can reduce *E*(*T*_*c*_)/*E*(*T*_*trip*,*non*−*VDRN*_) to a minimum of about 12 (Fig 7c). Since a minimum value of 12 is obtained in the worst-case situation, where the communication range is extremely small, a VDRN can reduce *E*(*T*_*c*_)/*E*(*T*_*trip*,*non*−*VDRN*_) to at least 12 by making drones pass each other more frequently. The trade-off for decreasing *E*(*T*_*c*_)/*E*(*T*_*trip*,*non*−*VDRN*_) using a VDRN is an increase in the mean travel time. For example, if we reduce *E*(*T*_*c*_)/*E*(*T*_*trip*,*non*−*VDRN*_) to 20 or 12, the mean travel time becomes 1.6 or 1.7 times longer (Fig 7c). However, if the purpose of the drone’s movement is not time-sensitive, such as information delivery in DTNs, such an increase in travel time is probably acceptable.

On the other hand, if we reduce *E*(*T*_*c*_)/*E*(*T*_*trip*,*non*−*VDRN*_) to 20 or 12 by increasing the communication range, then the communication range has to be about 3 or 4 times larger, as shown in Fig 6. Achieving a communication range that is 3 or 4 times larger is sometimes complicated due to power consumption or equipment limitations.

**Fig 9:**
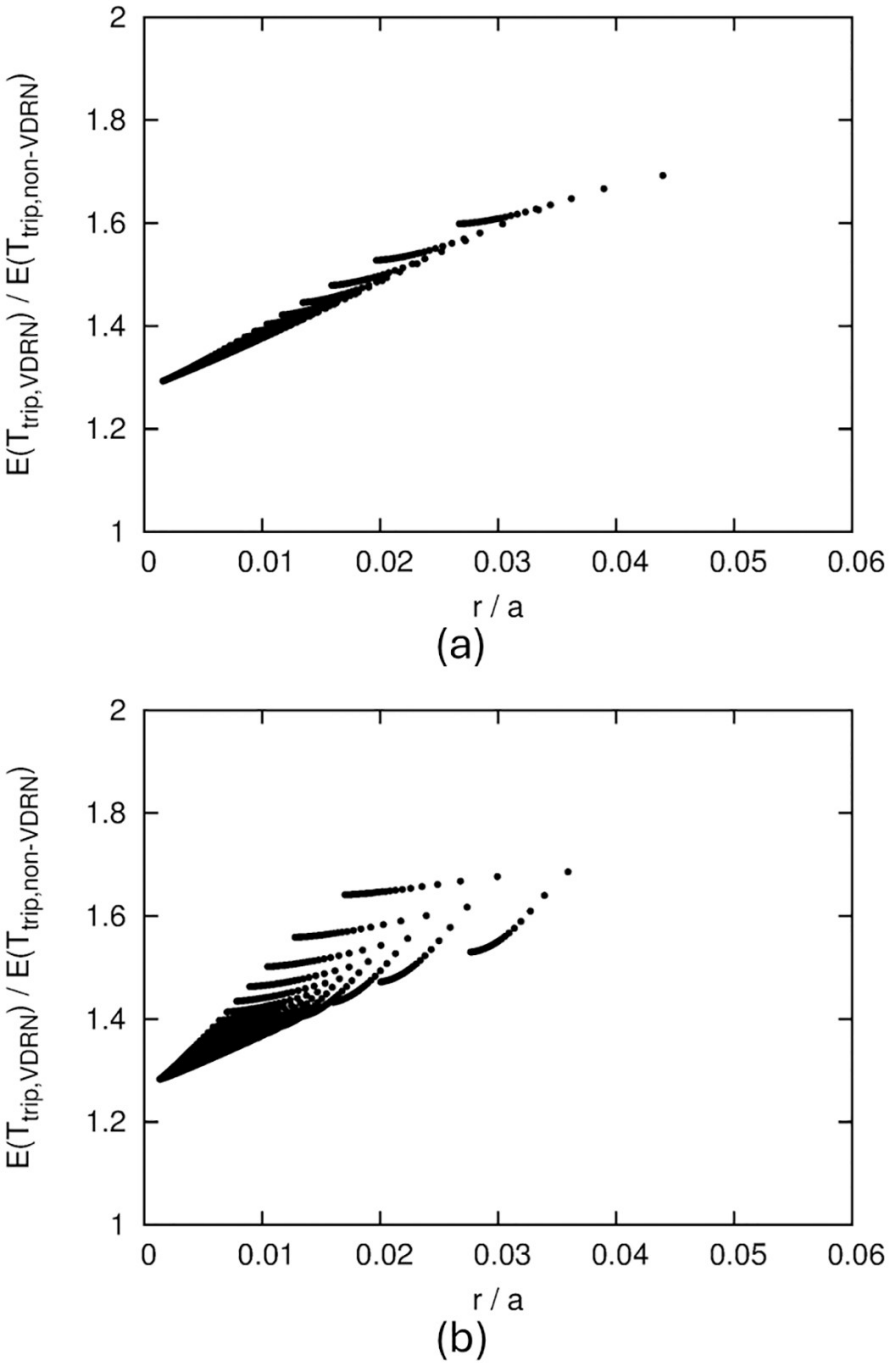
Relation between *r*/*a* for a non-VDRN and *E*(*T*_*trip*,*VDRN*_)/*E*(*T*_*trip*,*non*−*VDRN*_) for a VDRN, which achieve the same value of *E*(*T*_*c*_)/*E*(*T*_*trip*,*non*−*VDRN*_). (a) *b*/*a* = 1, (b) *b*/*a* = 0.7.

In Fig 9a, we show a relation indicating that an increase in communication range corresponds to longer mean travel time. This figure shows the relation between *r*/*a* for a non-VDRN and *E*(*T*_*trip*,*VDRN*_)/*E*(*T*_*trip*,*non*−*VDRN*_) for a VDRN, which achieve the same value of *E*(*T*_*c*_)/*E*(*T*_*trip*,*non*−*VDRN*_). The figure was obtained from Figs 6 and 7c and by numerically solving *E*(*T*_*c*,*VDRN*_) = *E*(*T*_*c*,*non*−*VDRN*_) with respect to *r*/*a* from the *E*(*T*_*c*,*VDRN*_) and *E*(*T*_*c*,*non*−*VDRN*_) formulas. These *r*/*a* and *E*(*T*_*trip*,*VDRN*_)/*E*(*T*_*trip*,*non*−*VDRN*_) values have a nearly linear relationship, suggesting that the ratio is nearly constant between the increases in *r*/*a* and *E*(*T*_*trip*,*VDRN*_)/*E*(*T*_*trip*,*non*−*VDRN*_) to reduce *E*(*T*_*c*_)/*E*(*T*_*trip*,*non*−*VDRN*_) by the same amount.

Fig 9b shows the results for *b*/*a* = 0.7, suggesting the same tendency as for *b*/*a* = 1.

### 5.4 Effect of communication range on performance of VDRN

In this paper, we evaluate the VDRN under the extreme worst-case scenario, where the communication range is extremely small and drones can only communicate if they pass each other on the VDRN. This is done to clarify the basic performance of VDRN. The theoretical analysis was made in this situation. In this section, we evaluate *E*(*T*_*c*,*VDRN*_)/*E*(*T*_*trip*,*non*−*VDRN*_) when the communication range of drones is larger than the above situation, as a reference evaluation. Since theoretical analysis for this case is difficult, we use computer simulation.

The parameters for the simulation are summarized in Table 3. The simulator was originally made by the authors using C#. In the simulation, two drones are deployed in the *a* × *b* rectangular service area and move independently according to the assumptions in Sections 3.1 and 3.3. We observe whether the drones are within communication range of each other at a constant time interval of Δ*t* = 0.1 s. Then we calculate the time intervals at which a drone enters the communication range of another drone, and we calculate its mean value as *E*(*T*_*c*_).

**Table 3:** Parameters used in simulation for VDRN.

Parameter	Value
*a*	10 km
*b*	10 km, 7 km
*m*	2
*v*	36 km/h
*r*	1 m
Simulation time	10^8^ s

**Fig 10:**
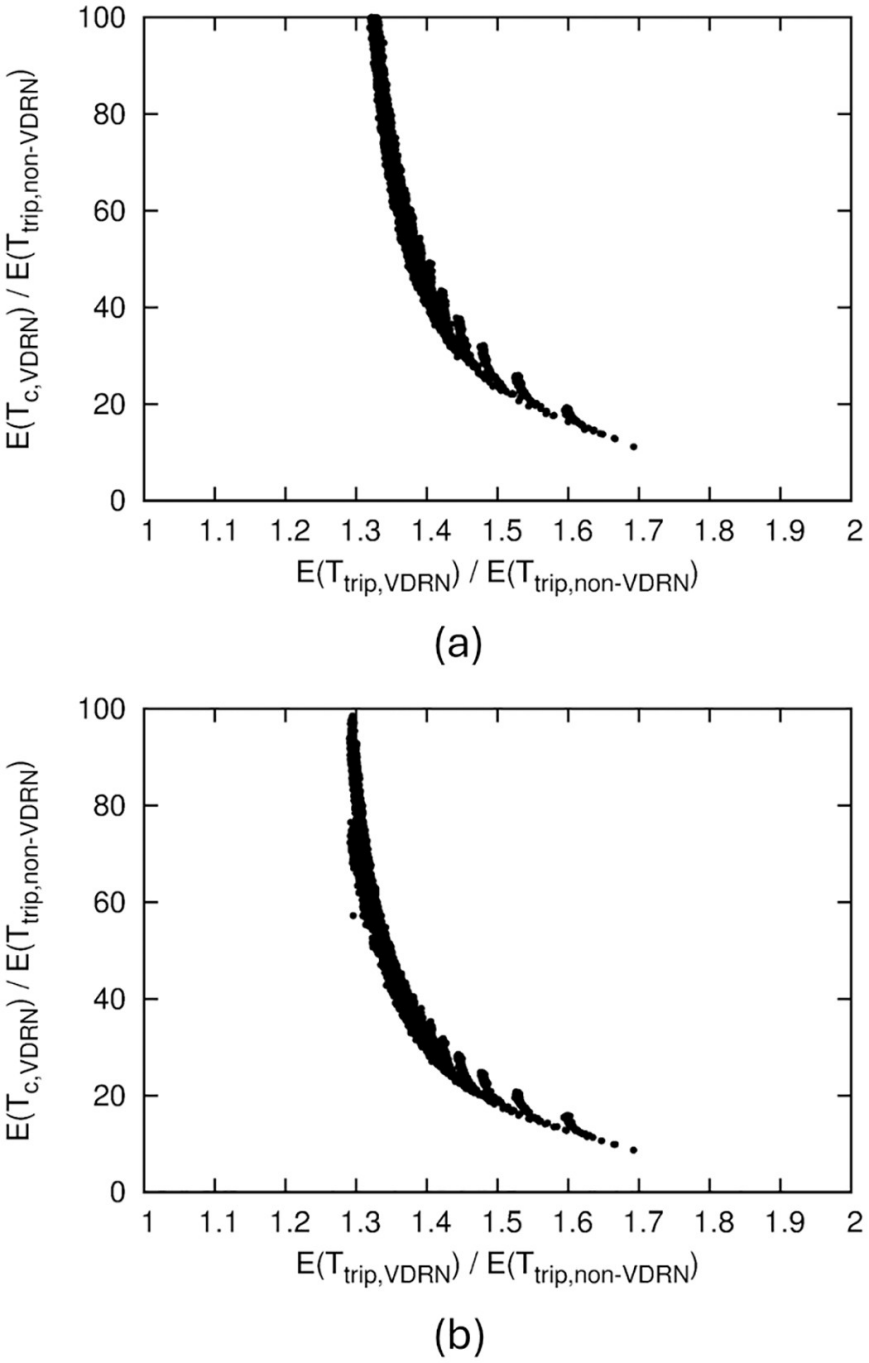
Simulation results of *E*(*T*_*c*,*VDRN*_)/*E*(*T*_*trip*,*non*−*VDRN*_) for a larger communication range, where *b*/*a* = 1. (a) *r* = 10 m, (b) *r* = 100 m.

The results for *b*/*a* = 1 are shown in Fig 10. In Fig 10a and 10b, *r* = 10 m and *r* = 100 m, respectively. The *r* = 10 m case assumes a very small communication range, while the *r* = 100 m case assumes a relatively large transmission range, such as Wireless LAN. The results for *r* = 10 m show that the values are almost the same as those in Fig 7c. This means that with a transmission range as small as *r* = 10 m, communication is nearly impossible except when drones pass each other on the VDRN. The high degree of agreement between the simulation results and the theoretical values in Fig 7c also confirms the validity of the theoretical analysis in this paper.

Next, the results for *r* = 100 m show that *E*(*T*_*c*,*VDRN*_)/*E*(*T*_*trip*,*non*−*VDRN*_) is much smaller than in Fig 7c. For example, the minimum value of *E*(*T*_*c*,*VDRN*_)/*E*(*T*_*trip*,*non*−*VDRN*_) for *r* = 100 m is about 8.7, while in Fig 7c it was about 12. This means we can use the theoretical formula of *E*(*T*_*c*,*VDRN*_), derived in this paper, as a safe-side evaluation.

**Fig 11:**
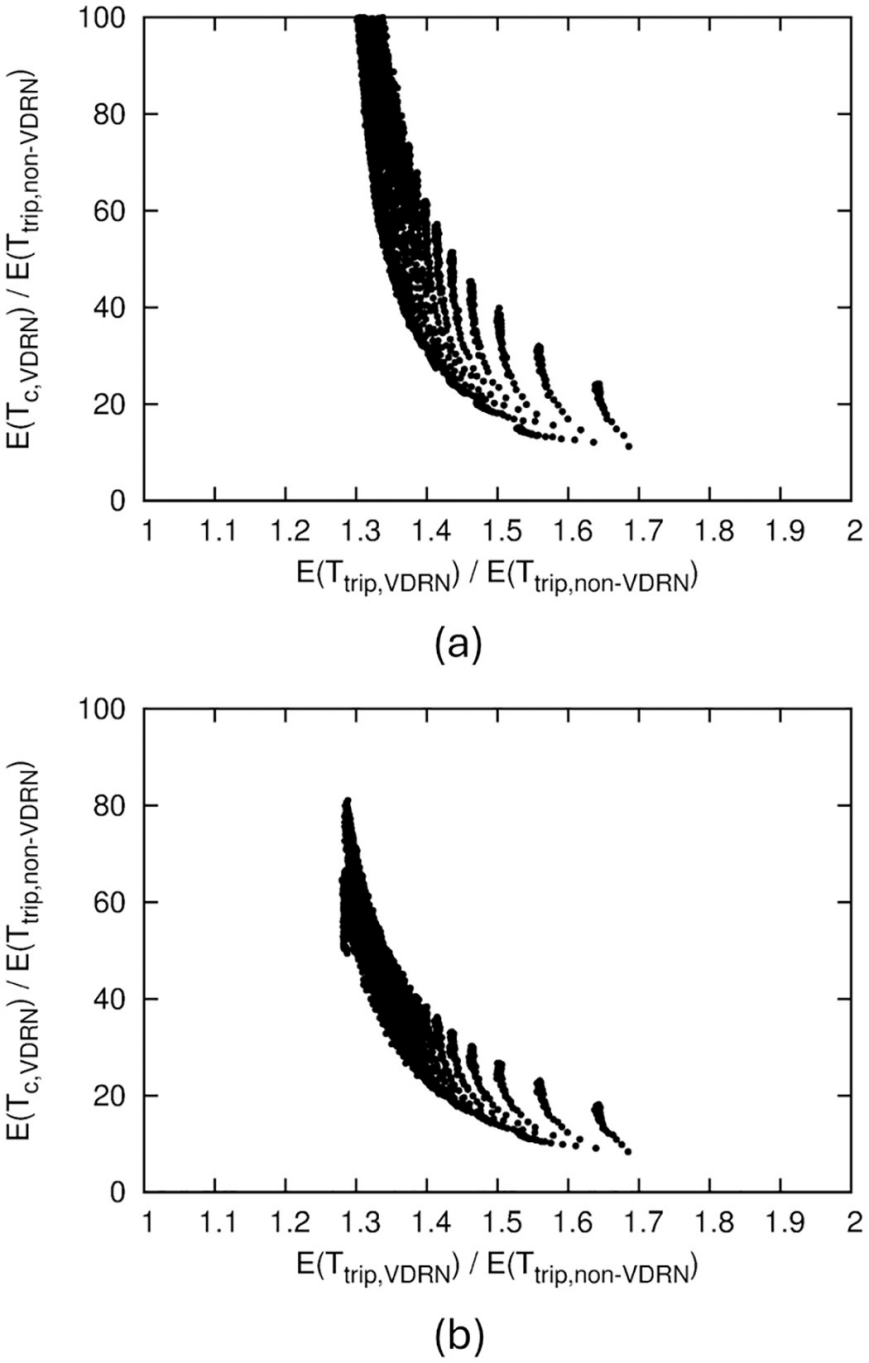
Simulation results of *E*(*T*_*c*,*VDRN*_)/*E*(*T*_*trip*,*non*−*VDRN*_) for a larger communication range, where *b*/*a* = 0.7. (a) *r* = 10 m, (b) *r* = 100 m.

The results for *b*/*a* = 0.7 are shown in Fig 11, and they show the same tendency as that for *b*/*a* = 1.

## 6 Conclusions

We described the establishment of a virtually deployed road network (VDRN) as a way to facilitate direct communication between drones and investigated the contact performance between them as they move distributedly along a VDRN. We established a VDRN with a lattice structure in a rectangular service area and clarified the trade-off between the mean contact interval and the mean travel time through theoretical analysis. We also theoretically analyzed these metrics for the case without a VDRN (non-VDRN), where drones moved directly to their destinations without detours, and showed the effect of the communication range on these metrics. From the numerical results, we clarified the amount of increase in the mean travel time required to achieve the same level of mean contact interval as that possible by increasing the communication range.

Our future work includes analyzing VDRNs with shapes other than a lattice structure. Achieving automatic VDRN generation and distribution is another important task for future work.

## 7 Appendix

### A Approximate analysis of *f*_Δ*X*,Δ*Y*_(*x*, *y*)

In general, Δ*X* and Δ*Y* are not independent because *X*_1_ and *Y*_1_ are not independent and *X*_2_ and *Y*_2_ are not independent. A previous report [23] showed that we can approximately assume that *X*_*i*_ and *Y*_*i*_ are independent in a square service area. We extend this idea to a rectangular service area and approximately assume that *X*_*i*_ and *Y*_*i*_ are independent. From this assumption, *X*_1_, *Y*_1_, *X*_2_, and *Y*_2_ are independent of each other because *X*_1_ and *Y*_1_ are independent of *X*_2_ and *Y*_2_ from the assumption that *n*_1_ and *n*_2_ move independently. Accordingly, Δ*X* and Δ*Y* are independent of each other:

$$\begin{array}{c} f_{\Delta X,\Delta Y}\left(x,y\right)=f_{\Delta X}\left(x\right)f_{\Delta Y}\left(y\right). \end{array}$$ (18)

*f*_Δ*X*_(*x*) and *f*_Δ*Y*_(*y*) can be represented as follows from the definitions of Δ*X* and Δ*Y*:

$$\begin{array}{c} f_{\Delta X}\left(x\right)=\int_{0}^{a}f_{X_{1}}\left(x_{1}\right)\left\{f_{X_{2}}\left(x_{1}-x\right)+f_{X_{2}}\left(x_{1}+x\right)\right\}dx_{1}, \end{array}$$ (19)

$$\begin{array}{c} f_{\Delta Y}\left(y\right)=\int_{0}^{b}f_{Y_{1}}\left(y_{1}\right)\left\{f_{Y_{2}}\left(y_{1}-y\right)+f_{Y_{2}}\left(y_{1}+y\right)\right\}dy_{1}, \end{array}$$ (20)

where $f_{X_{i}}\left(x\right)$ and $f_{Y_{i}}\left(y\right)$ are the probability density functions of *X*_*i*_ and *Y*_*i*_. To compute Eqs (19) and (20), we next analyze $f_{X_{i}}\left(x\right)$ and $f_{Y_{i}}\left(y\right)$.

Since precisely analyzing $f_{X_{i}}\left(x\right)$ and $f_{Y_{i}}\left(y\right)$ is also difficult due to the dependence of *X*_*i*_ and *Y*_*i*_, we analyze them approximately. The same previous report [23] also argued that for a square service area of [0, *a*] × [0, *a*], each $f_{X_{i}}\left(x\right)$ and $f_{Y_{i}}\left(y\right)$ can be approximated by the probability density function of the location of a point moving according to a one-dimensional RWP on [0, *a*]. We extend this idea to the case of a rectangular service area and approximately analyze $f_{X_{i}}\left(x\right)$ and $f_{Y_{i}}\left(y\right)$ as the probability density functions of the locations of points moving, again based on a one-dimensional RWP, on [0, *a*] and [0, *b*], respectively. These probability density functions can be precisely analyzed [23]:

$$\begin{array}{c} f_{X_{i}}\left(x\right)=\{\begin{array}{cc} \frac{6x(a-x)}{a^{3}}, & 0\leq x\leq a,\\ 0, & \text{otherwise,} \end{array} \end{array}$$ (21)

$$\begin{array}{c} f_{Y_{i}}\left(y\right)=\{\begin{array}{cc} \frac{6y(b-y)}{b^{3}}, & 0\leq y\leq b,\\ 0, & \text{otherwise.} \end{array} \end{array}$$ (22)

By substituting Eqs (21) and (22) into Eqs (19) and (20), we have Eqs (5) and (6). Finally, by substituting Eqs (5) and (6) into Eq (18), we approximately compute *f*_Δ*X*,Δ*Y*_(*x*, *y*).

### B Approximate analysis of *E*(*T*_*cc*_)

Since precisely analyzing *E*(*T*_*cc*_) is difficult, we examine it approximately as follows. As an approximation, we suppose that both *n*_1_ and *n*_2_ move uniformly in random directions on a sufficiently wide plane, neglecting the effects of positional bias due to RWP as well as the effects of the edge of the service area. In such a situation, we focus on the moment when *n*_1_ and *n*_2_ enter each other’s communication range. Since *E*(*T*_*cc*_) only depends on the relative motion of *n*_1_ and *n*_2_, we consider the relative motion of *n*_2_ with respect to *n*_1_.

For any time *t* and very small time duration *dt*, let *A* be the event at which *n*_1_ and *n*_2_ enter each other’s communication range between times *t* and *t* + *dt*. Let Θ be a random variable representing a relative angle in the direction of the motion of *n*_2_ with respect to the direction of the motion of *n*_1_, where 0 ≤ Θ < 2*π*. By using them, *E*(*T*_*cc*_) can be represented:

$$\begin{array}{c} E\left(T_{cc}\right)=\int_{0}^{2\pi }f_{\Theta }\left(\theta |A\right)E\left(T_{cc}|A,\Theta =\theta \right)d\theta , \end{array}$$ (23)

where *f*_Θ_(*θ*|*A*) is the probability density function of Θ given that *A* occurs. To compute this, we next analyze *f*_Θ_(*θ*|*A*) and *E*(*T*_*cc*_|*A*, Θ = *θ*).

First, from the formula for the conditional probability density function, we have

$$\begin{array}{c} f_{\Theta }\left(\theta |A\right)=\frac{f_{\Theta }\left(\theta \right)\text{Pr}\left(A|\Theta =\theta \right)}{\int_{0}^{2\pi }f_{\Theta }\left(\theta^{\prime }\right)\text{Pr}\left(A|\Theta =\theta^{\prime }\right)d\theta^{\prime }}. \end{array}$$ (24)

Here, the frequency of contact is proportional to the relative speed of *n*_1_ and *n*_2_; therefore, we have Pr(*A*|Θ = *θ*) ∝ *v*_*r*_(*θ*), where *v*_*r*_(*θ*) is the relative speed of *n*_2_ with respect to *n*_1_. *v*_*r*_(*θ*) can be computed as

$$\begin{array}{c} v_{r}\left(\theta \right)=\sqrt{{\left(v\;\text{cos}\;\theta -v\right)}^{2}+{\left(v\;\text{sin}\;\theta \right)}^{2}}=2v\;\text{sin}\;\frac{\theta }{2}. \end{array}$$ (25)

Since *n*_1_ and *n*_2_ move in random directions independently of each other, Θ is uniformly distributed in [0, 2*π*), and its probability density function is *f*_Θ_(*θ*) = 1/(2*π*). From these equations, we have

$$\begin{array}{c} f_{\Theta }\left(\theta |A\right)=\frac{\frac{1}{2\pi }v_{r}\left(\theta \right)}{\int_{0}^{2\pi }\frac{1}{2\pi }v_{r}\left(\theta^{\prime }\right)d\theta^{\prime }}=\frac{1}{4}\;\text{sin}\;\frac{\theta }{2}. \end{array}$$ (26)

**Fig 12:**
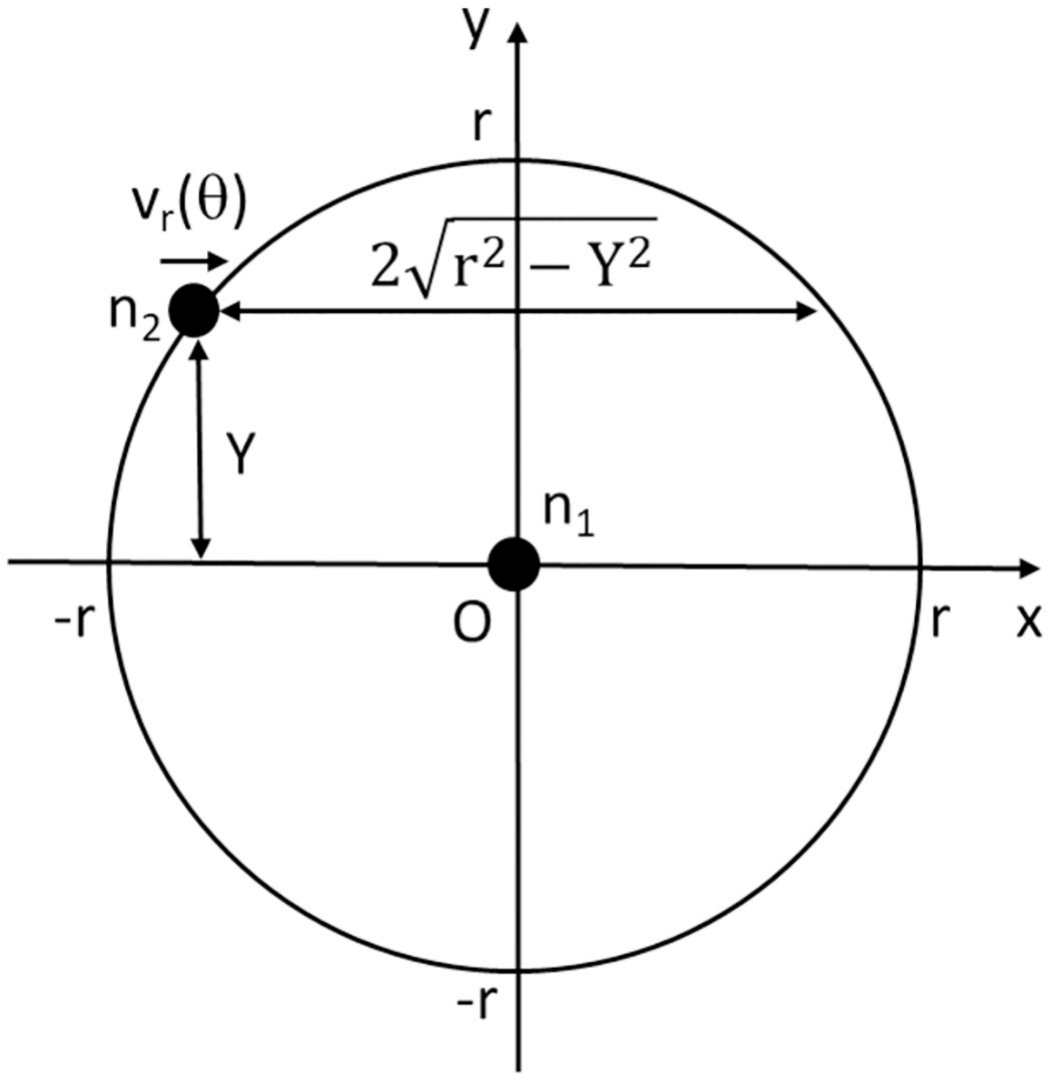
Situation where *A* occurs and Θ = *θ*.

Next we analyze *E*(*T*_*cc*_|*A*, Θ = *θ*). Consider a situation where *A* occurs and Θ = *θ*. As mentioned above, we only consider the relative motion of *n*_1_ and *n*_2_. We regard *n*_1_ as stationary at the origin and consider a situation where *n*_2_ enters the communication range of *n*_1_ (Fig 12). If *n*_2_ moves in the x-axis direction without loss of generality, the y-axis value of *n*_2_, denoted as *Y*, clearly obeys a uniform distribution of [−*r*, *r*]. Furthermore, *E*(*T*_*cc*_|*A*, Θ = *θ*, *Y* = *y*) can be computed by $2\sqrt{r^{2}-y^{2}}/v_{r}\left(\theta \right)$ from Fig 12. Therefore, we have

$$\begin{array}{c} E\left(T_{cc}|A,\Theta =\theta \right)=\frac{1}{2r}\int_{-r}^{r}E\left(T_{cc}|A,\Theta =\theta ,Y=y\right)dy=\frac{\pi r}{2v_{r}\left(\theta \right)}. \end{array}$$ (27)

By substituting Eqs (26) and (27) into Eq (23), *E*(*T*_*cc*_) can be computed by Eq (7).

### C Analysis of Pr(*A*_1_(*PQ*))

Since directly analyzing Pr(*A*_1_(*PQ*)) is difficult, we analyze it through event *A*_2_(*c*_*S*_, *c*_*D*_) as follows. *A*_2_(*c*_*S*_, *c*_*D*_) is an event such that at any given time a drone is on a path with starting intersection *c*_*S*_ and destination intersection *c*_*D*_. Consequently, Pr(*A*_1_(*PQ*)) can be represented as

$$\begin{array}{c} \text{Pr}\left(A_{1}\left(PQ\right)\right)=\sum_{c_{S}\in C}\sum_{c_{D}\in C}\text{Pr}\left(A_{2}\left(c_{S},c_{D}\right)\right)\text{Pr}\left(A_{1}\left(PQ\right)|A_{2}\left(c_{S},c_{D}\right)\right). \end{array}$$ (28)

Pr(*A*_2_(*c*_*S*_, *c*_*D*_)) can be analyzed as follows. Let *ℓ*(*c*_*S*_, *c*_*D*_) be the length of a path from *c*_*S*_ to *c*_*D*_. It is computed as *ℓ*(*c*_*S*_, *c*_*D*_) = |*i*_*S*_ − *i*_*D*_|*d*_*x*_ + |*j*_*S*_ − *j*_*D*_|*d*_*y*_, where $c_{S}=c_{i_{S},j_{S}}$ and $c_{D}=c_{i_{D},j_{D}}$. Obviously, Pr(*A*_2_(*c*_*S*_, *c*_*D*_)) is proportional to *ℓ*(*c*_*S*_, *c*_*D*_). Furthermore, *A*_2_(*c*_*S*_, *c*_*D*_) occurs only if the drone is on a VDRN. From the analysis in Section 4.3, the probability that a drone is on a VDRN at any given time is *E*(*L*_*L*_)/{*E*(*L*_*L*_) + *E*(*L*_*NL*_)}. From these facts, we have

$$\begin{array}{c} \text{Pr}\left(A_{2}\left(c_{S},c_{D}\right)\right)=\frac{E(L_{L})}{E\left(L_{L}\right)+E\left(L_{NL}\right)}\cdot \frac{\ell (c_{S},c_{D})}{\sum_{c_{S}^{\prime }\in C}\sum_{c_{D}^{\prime }\in C}\ell \left(c_{S}^{\prime },c_{D}^{\prime }\right)}. \end{array}$$ (29)

**Fig 13:**
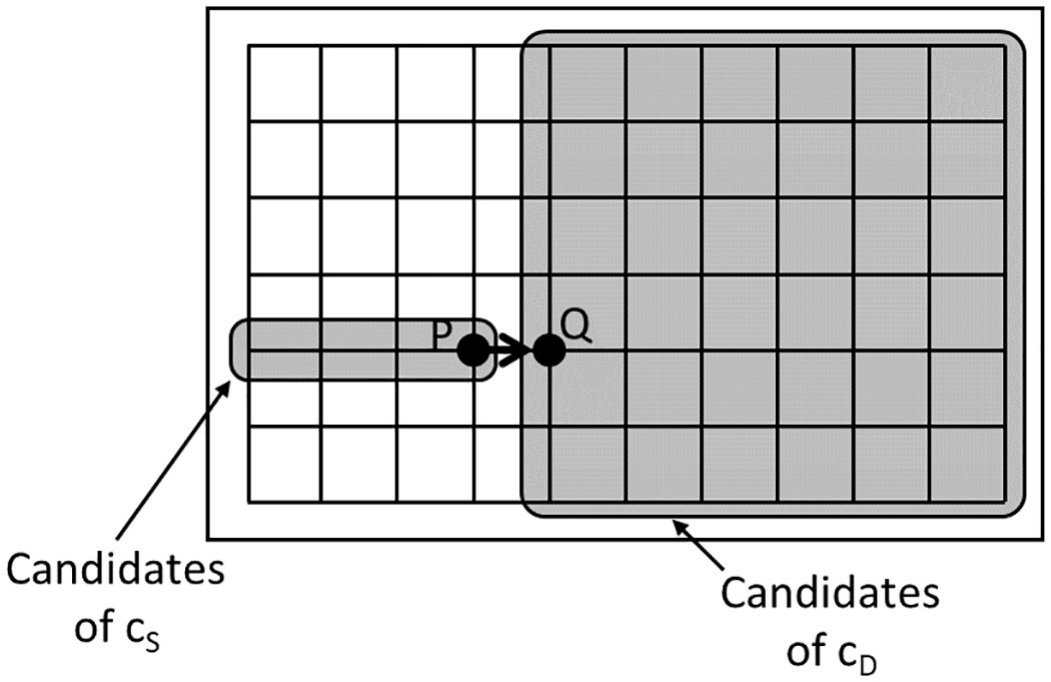
Example of *C*_*E*_(*PQ*).

**Fig 14:**
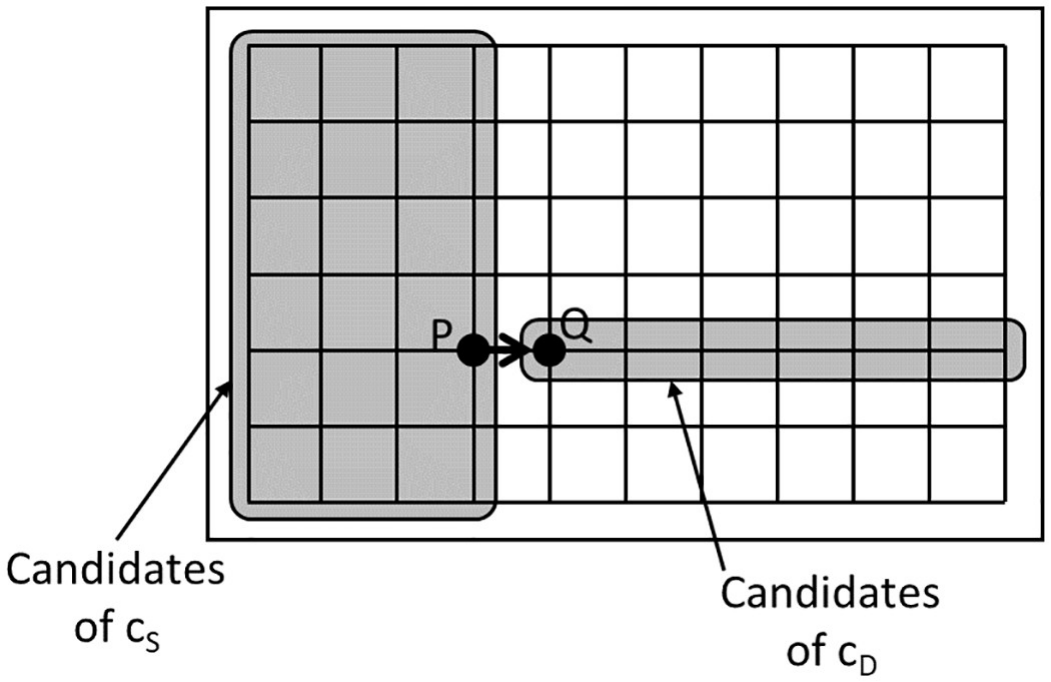
Example of $C_{E}^{\prime }\left(PQ\right)$.

Pr(*A*_1_(*PQ*)|*A*_2_(*c*_*S*_, *c*_*D*_)) can be analyzed as follows. If a path from *c*_*S*_ to *c*_*D*_ includes link PQ, then Pr(*A*_1_(*PQ*)|*A*_2_(*c*_*S*_, *c*_*D*_)) = *ℓ*(*P*, *Q*)/*ℓ*(*c*_*S*_, *c*_*D*_). Otherwise, Pr(*A*_1_(*PQ*)|*A*_2_(*c*_*S*_, *c*_*D*_)) = 0. Note that *ℓ*(*P*, *Q*) = *d*_*x*_ if *PQ* ∈ *S*_*E*_ ∪ *S*_*W*_, and *ℓ*(*P*, *Q*) = *d*_*y*_ if *PQ* ∈ *S*_*N*_ ∪ *S*_*S*_. Whether a path from *c*_*S*_ to *c*_*D*_ includes link PQ depends on whether the drone first moves horizontally or vertically. Let *A*_3_ be an event in which the drone first moves horizontally to travel from *c*_*S*_ to *c*_*D*_. From the assumption, $\text{Pr}\left(A_{3}\right)=\text{Pr}\left(\bar{A_{3}}\right)=1/2$. First, suppose that *PQ* ∈ *S*_*E*_ and *A*_3_ occurs. In this case, a path from *c*_*S*_ to *c*_*D*_ includes link PQ if and only if (*c*_*S*_, *c*_*D*_) ∈ *C*_*E*_(*PQ*), where $C_{E}\left(PQ\right)=\left\{\left(c_{i_{S},j_{S}},c_{i_{D},j_{D}}\right)|0\leq i_{S}\leq i_{P},j_{S}=j_{P},i_{P}+1\leq i_{D}\leq N_{x},0\leq j_{D}\leq N_{y}\right\}$ and where $P=c_{i_{P},j_{P}}$ (Fig 13). Next, suppose that *PQ* ∈ *S*_*E*_ and $\bar{A_{3}}$ occurs. In this case, a path from *c*_*S*_ to *c*_*D*_ includes link PQ if and only if $\left(c_{S},c_{D}\right)\in C_{E}^{\prime }\left(PQ\right)$, where $C_{E}^{\prime }\left(PQ\right)=\left\{\left(c_{i_{S},j_{S}},c_{i_{D},j_{D}}\right)|0\leq i_{S}\leq i_{P},0\leq j_{S}\leq N_{y},i_{P}+1\leq i_{D}\leq N_{x},j_{D}=j_{P}\right\}$ and where $P=c_{i_{P},j_{P}}$ (Fig 14). To summarize the above, for *PQ* ∈ *S*_*E*_, we have

$$\begin{array}{cc} & \text{Pr}(A_{1}\left(PQ\right)|A_{2}\left(c_{S},c_{D}\right))\\ & =\text{Pr}\left(A_{3}\right)\text{Pr}\left(A_{1}\left(PQ\right)|A_{2}\left(c_{S},c_{D}\right),A_{3}\right)+\text{Pr}\left(\bar{A_{3}}\right)\text{Pr}\left(A_{1}\left(PQ\right)|A_{2}\left(c_{S},c_{D}\right),\bar{A_{3}}\right), \end{array}$$ (30)

$$\begin{array}{c} \text{Pr}\left(A_{1}\left(PQ\right)|A_{2}\left(c_{S},c_{D}\right),A_{3}\right)=\{\begin{array}{cc} \frac{d_{x}}{\ell (c_{S},c_{D})}, & \left(c_{S},c_{D}\right)\in C_{E}\left(PQ\right),\\ 0, & \text{otherwise}, \end{array} \end{array}$$ (31)

$$\begin{array}{c} \text{Pr}\left(A_{1}\left(PQ\right)|A_{2}\left(c_{S},c_{D}\right),\bar{A_{3}}\right)=\{\begin{array}{cc} \frac{d_{x}}{\ell (c_{S},c_{D})}, & \left(c_{S},c_{D}\right)\in C_{E}^{\prime }\left(PQ\right),\\ 0, & \text{otherwise}. \end{array} \end{array}$$ (32)

By substituting Eqs (29), (30), (31), and (32) into Eq (28), we can compute Pr(*A*_1_(*PQ*)) for *PQ* ∈ *S*_*E*_ by Eq (15). In the same manner, we can compute Pr(*A*_1_(*QP*)) for *QP* ∈ *S*_*W*_ by Eq (15) and Pr(*A*_1_(*PQ*)) for *PQ* ∈ *S*_*N*_ and Pr(*A*_1_(*QP*)) for *QP* ∈ *S*_*S*_ by Eq (16).
